# Gold-catalyzed intermolecular hydroamination of allenes with sulfonamides

**DOI:** 10.3762/bjoc.9.117

**Published:** 2013-05-29

**Authors:** Chen Zhang, Shao-Qiao Zhang, Hua-Jun Cai, Dong-Mei Cui

**Affiliations:** 1 School of Pharmaceutical Sciences, Zhejiang University, Hangzhou 310058, PR China; 2 College of Pharmaceutical Science, Zhejiang University of Technology, Hangzhou 310014, PR China

**Keywords:** allene, gold catalysis, hydroamination, *N*-sulfonyl, selectivity, sulfonamide

## Abstract

A co-catalyst of (PPh_3_)AuCl/AgOTf for the intermolecular hydroamination of allenes with sulfonamides is shown. The reaction proceeded smoothly under mild conditions for differently substituted allenes giving *N*-allylic sulfonamides in good yields with high regioselectivity and *E*-selectivity.

## Introduction

Hydroamination of an N–H bond across a C–C unsaturated bond represents one of the most effective and atom-economical methods to prepare amine derivatives [1–5]. In the case of using allenes, this reaction can lead to allylamines, which are invaluable precursors for the synthesis of natural products and other potentially biologically relevant substances [6]. In the literature, a wide range of catalytic intramolecular hydroaminations of allenes are known, but only a small number of intermolecular hydroamination reactions are reported [7–15]. More recently, Au(I), Au(III), Pt(II) and Rh(I) have been used for the intermolecular hydroamination of allenes with secondary alkylamines, ammonia, or carboxamide [7,16–24]. Although some of these advances have been efficiently made in hydroamination, many require extreme and extended reaction conditions. Thus, development of these reactions is still needed. Recently, Yamamoto and co-workers reported the Pd(0)-catalyzed intermolecular hydroamination of allenes with sulfonamides [25]. In this paper, we wish to develop a gold(I)-complex-catalyzed addition of sulfonamides as the amine partner to allenes to synthesize *N*-allylic sulfonamides with high regio- and stereoselectivity.

## Results and Discussion

As part of our ongoing studies on metal-catalyzed reactions, we have reported the hydroalkoxylation of allenes with alcohols and hydroamination of alkynes with sulfonamides in the presence of gold catalysts [26–28]. On the basis of these studies, in an initial experiment, 1-phenyl-1,2-propadiene (**1a**) (1.5 mmol) was treated with 4-methylbenzenesufonamide (**2a**) (0.5 mmol) in the presence of 2 mol % of (PPh_3_)AuCl and 8 mol % of AgOTf in dioxane at 70 °C efficiently to form linear adduct **3a** in 43% yield (Table 1, entry 1). Different solvents were screened, and dioxane was found to be the most suitable one (Table 1, entries 2–4). Decreasing the amount of AgOTf resulted in lower yields (Table 1, entry 7). We were pleased to find that efficient hydroamination was realized at rt and led to a 91% yield of **3a** with good regioselectivity and high *E*-selectivity (Table 1, entry 6). Other possible isomers could not be detected. As Ag catalysts, other salts were also screened, AgBF_4_ was ineffective. With AgSbF_6_ or AgNTf_2_, the reaction took place and gave adducts in 33% and 47% yield (Table 1, entries 8 and 9). The use of the gold alone gave a lower yield, and the reaction did not proceed in the absence of gold or through the use of TfOH (entries 11–14). Finally, we determined the optimal conditions as 5 mol % of (PPh_3_)AuCl and 8 mol % of AgOTf in dioxane at rt (Table 1, entry 6).

**Table 1:** Catalytic hydroamination of **1a** and **2a**.^a^

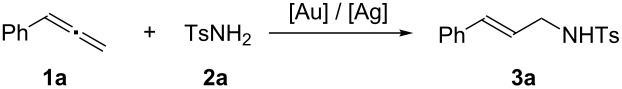						

Entry	[Au] (mol %)	[Ag] (mol %)	Solvent	Time (h)	Temp. (°C)	Yield (%)^b^

1	(PPh_3_)AuCl (2)	AgOTf (8)	dioxane	4	70	43
2	(PPh_3_)AuCl (2)	AgOTf (8)	THF	6	70	28
3	(PPh_3_)AuCl (2)	AgOTf (8)	toluene	8	70	32
4	(PPh_3_)AuCl (2)	AgOTf (8)	(CH_2_Cl)_2_	7	70	33
5	(PPh_3_)AuCl (5)	AgOTf (8)	dioxane	4	70	59
6	(PPh_3_)AuCl (5)	AgOTf (8)	dioxane	16	rt	91
7	(PPh_3_)AuCl (5)	AgOTf (5)	dioxane	16	rt	80
8	(PPh_3_)AuCl (5)	AgSbF_6_ (8)	dioxane	24	rt	33
9	(PPh_3_)AuCl (5)	AgNTf_2_ (8)	dioxane	24	rt	47
10	Au(NHC)Cl (5)	AgOTf (8)	dioxane	16	rt	46
11	0	0	dioxane	16	rt	0
12	(PPh_3_)AuCl (5)	0	dioxane	16	rt	26
13	0	AgOTf (8)	dioxane	16	rt	0
14	0	TfOH (8)	dioxane	16	rt	0

^a^All reactions were performed with 0.8 mmol of **1a**, 0.4 mmol of **2a**, 0–5 mol % of (PPh_3_)AuCl, and 0–8 mol % of AgOTf. ^b^Isolated yields.

To further assess the scope of this process, we first examined the hydroamination of **1a** with several sulfonamides. Benzenesulfonamides containing *p*-Br or *p*-Cl groups on the benzene ring were tolerated for the reaction, obtaining the corresponding adducts **3d** and **3e** in 54 and 72% yields, respectively (Table 2, entries 4–5). Under the same reaction conditions, the hydroamination of aliphatic sulfonamides took place smoothly to afford the corresponding *N*-allylic sulfonamide **3f** with 56% yield (Table 2, entry 6). We also used *N*-substituted sulfonamide **2g** as the amine partner. Although drastic conditions are required, the addition occurred to provide linear adduct **3g** in good yield (Table 2, entry 7). In all cases, the adduct was obtained with high selectivity.

**Table 2:** Hydroamination of **1a** with sulfonamide **2**.^a^

Entry	Sulfonamide	**2**	Product	**3**	Yield (%)^b^

1	TsNH_2_	**2a**	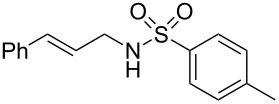	**3a**	91
2	PhSO_2_NH_2_	**2b**	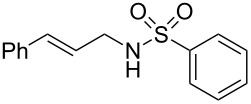	**3b**	76
3	*o*-Me-C_6_H_4_SO_2_NH_2_	**2c**	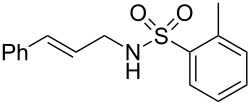	**3c**	60
4	*p*-Br-C_6_H_4_SO_2_NH_2_	**2d**	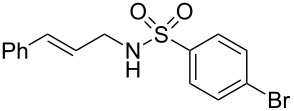	**3d**	54
5	*p*-Cl-C_6_H_4_SO_2_NH_2_	**2e**	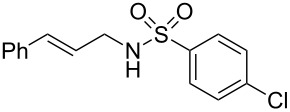	**3e**	72
6	MeSO_2_NH_2_	**2f**	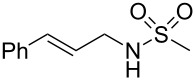	**3f**	56
7^c^	*n-*BuNHTs	**2g**	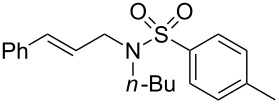	**3g**	79

^a^The reactions were performed with 0.8 mmol of allene **1a**, 0.4 mmol of **2**, 5 mol % of (Ph_3_P)AuCl and 8 mol % of AgOTf in 2 mL of dioxane at rt for 16 h. ^b^Isolated yield. ^c^At 100 °C for 8 h.

**Figure 1:**
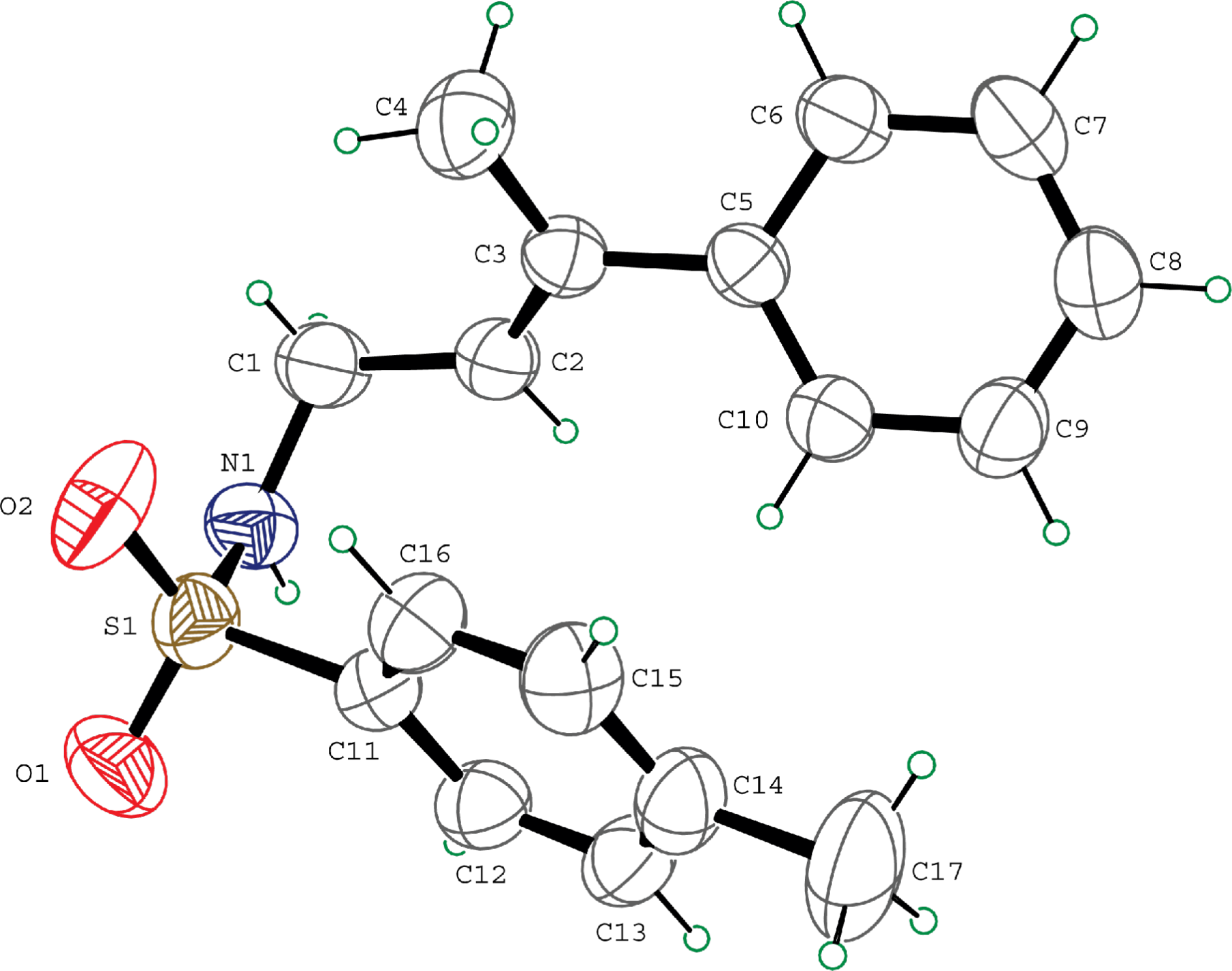
The X-ray structure of **3l**.

**Figure 2:**
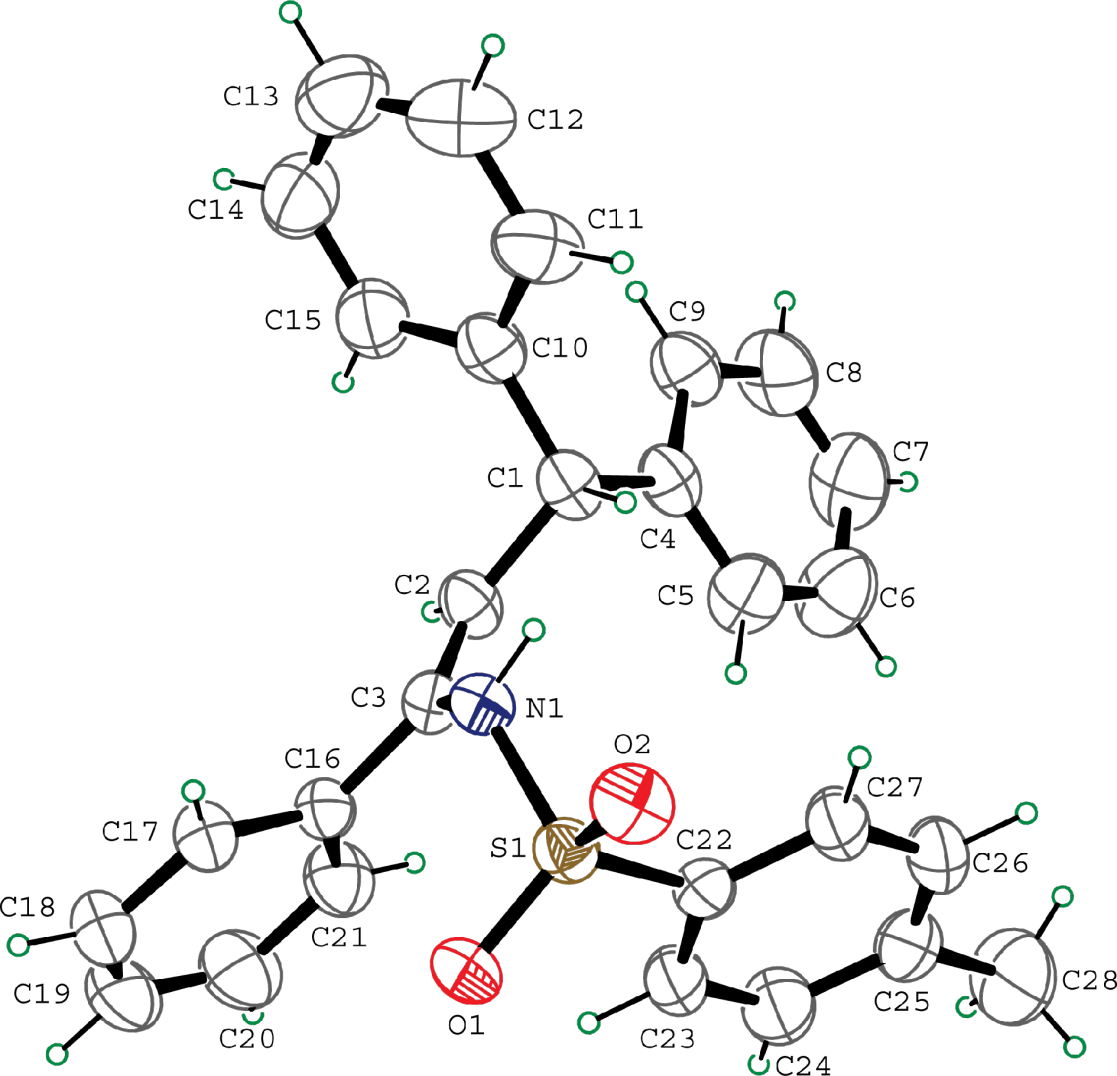
The X-ray structure of **3n**.

Various allenes were then examined, and aromatic rings of phenylallenes with either an electron-donating or an electron-withdrawing group gave good isolated yields of the corresponding adducts (Table 3, entries 1 and 2). Whereas hydroamination of the monosubstituted heteroaromatic allene **1d** also lead to the conversion into the expected addition product **3j**, hydroamination of the monoalkyl-substituted aliphatic allene **1e** formed a 71:29 mixture of linear product (**3ka**) and branch product (**3kb**) under the same conditions (Table 3, entry 4). Furthermore, disubstituted allenes also worked well. Differentially 1,1-disubstituted allene **1f** reacted with sulfonamide to afford *trans*-adducts **3l** with high selectivity (Table 3, entry 5). Single crystals of the compound **3l** suitable for X-ray crystallographic analysis were also obtained (Figure 1). This shows that **3l** is the *E* isomer, the sulfonamide carbon link being *trans* to the phenyl group (Figure 1). As for differentially 1,3- disubstituted allene **1g**, hydroamination took place with exclusive attack of sulfonamide at the more electron-rich allene terminus to afford the corresponding adduct **3m** with 68% yield and with high *E*-selectivity (Table 3, entry 6). In addition, hydroamination of trisubstituted allene **1h** took place to afford a different product (Table 3, entry 7). Single crystals of the compound **3n** suitable for X-ray crystallographic analysis were also obtained (Figure 2). This showed that **3n** is the *E* isomer, the sulfonamide being *trans* to the diphenylmethyl groups (Figure 2).

**Table 3:** Hydroamination of allenes **1** with **2a**.^a^

Entry	Allene	**1**	Product	**3**	Yield (%)

1	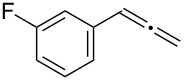	**1b**	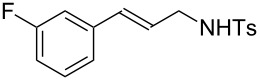	**3h**	82
2	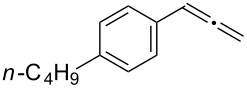	**1c**	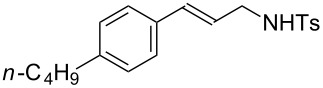	**3i**	72
3	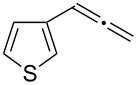	**1d**	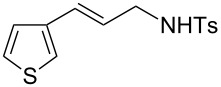	**3j**	60
4	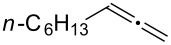	**1e**	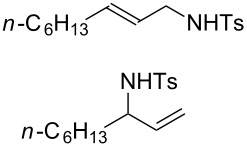	**3ka** **3kb**	48
5	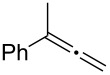	**1f**	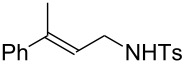	**3l**	67
6	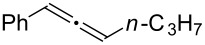	**1g**	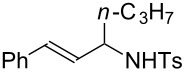	**3m**	68
7	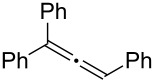	**1h**	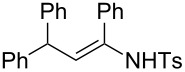	**3n**	31

^a^The reactions were performed with 0.8 mmol of allene **1**, 0.4 mmol of **2a**, 5 mol % of (Ph_3_P)AuCl and 8 mol % of AgOTf in 2 mL of dioxane at rt for 16 h. ^b^Isolated yield. ^c^**3ka**/**3kb** = 71:29.

**Scheme 1:**
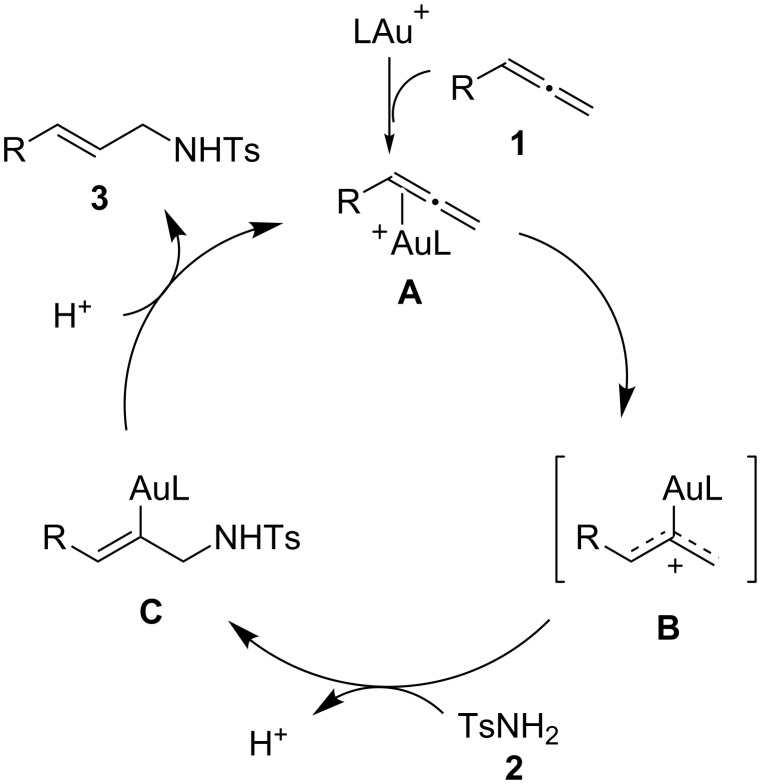
Proposed mechanism for the hydroamination of allenes.

The proposed mechanism of the gold-catalyzed hydroaamination of allenes is shown in Scheme 1 [2,7,29–36]. The gold cation coordinated with allene to form cationic Au(I)-allene complex **A**, and this leads to cationic gold(I) complex **B**. The sulfonamide attacks at the less-substituted terminus of intermediate **B** to form **C**. Protonolysis of the Au–C bond of **B** yields the allylic sulfonamide **3**, regenerating the gold complex. On the other hand, in comparison with phenyl-substituted allenes, for alkyl-substituted allene **1e**, a mixture of **3ka** and **3kb** was produced; although the details are unclear, perhaps due to electronic factors, the addition of sulfonamide also occurred at the more-hindered position of intermediate **B** to give **3ka** and **3kb**.

## Conclusion

In conclusion, we have successfully employed (PPh_3_)AuCl/AgOTf catalyzed intermolecular hydroamination of allenes with sulfonamides to produce *N*-allylic sulfonamide. This reaction takes place under mild conditions with effective and high regio- and stereoselectivity. Monosubstituted, 1,1- and 1,3-disubstituted, and trisubstituted allenes were well tolerated in the reaction.

## Experimental

**General Information**: Unless otherwise noted, materials were obtained from commercial suppliers and used without further purification. Allenes were prepared by procedures in the literature [37–39]. Thin-layer chromatography (TLC) was performed on glass plates coated with silica gel 60 F254 and visualized by UV light (254 nm). Column chromatography was performed with silica gel (mesh 300–400). Infrared (IR) spectra were obtained on a 370 FTIR spectrometer; absorptions are reported in cm^−1^. Mass spectra were obtained in the electron impact (EI) mode, and high-resolution mass spectra were measured on a high-resolution mass spectrometer (GCT Premier).

**General Procedure:** To a mixture of sulfonamide (0.4 mmol), PPh_3_AuCl (0.02 mmol), and AgOTf (0.032 mmol) in anhydrous 1,4-dioxane (2 mL) was added allene (0.8 mmol). The mixture was then sealed and stirred at room temperature until the starting sulfonamide was consumed as judged by TLC. The mixture was quenched with a saturated solution of NaHCO_3_ and then extracted with ethyl acetate (3 × 20 mL). The organic layer was washed with brine, dried over Na_2_SO_4_ and concentrated in vacuo. The residue was purified by column chromatography (silica gel) to yield the product in an analytically pure form.

## Supporting Information

File 1 Analytical and spectroscopic data for compounds **3a**–**3j**, **3ka**, **3kb** and **3l**–**3n**.
