# Supplementary material for: Gold-catalyzed intermolecular hydroamination of allenes with sulfonamides
Source: Beilstein J Org Chem. 2013 May 29;9:1045–50. doi: 10.3762/bjoc.9.117 (PMC3678397; doi:10.3762/bjoc.9.117)

Supporting Information

for

Gold-catalyzed intermolecular hydroamination of allenes with sulfonamides

Chen Zhang*[,1], Shao-Qiao Zhang[1], Hua-Jun Cai[2] and Dong-Mei Cui*[2]

Address: [1]School of Pharmaceutical Sciences, Zhejiang University, Hangzhou 310058, PR China and [2]College of Pharmaceutical Science, Zhejiang University of Technology, Hangzhou 310014, PR China

Email: Dong-Mei Cui - cuidongmei@zjut.edu.cn

* Corresponding author

Analytical and spectroscopic data for compounds 3a–3j, 3ka, 3kb and 3l–3n.

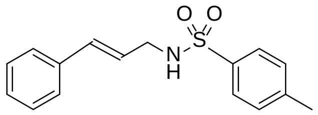

***N*-cinnamyl-4-methylbenzenesulfonamide** (**3a**) [1]: White solid; mp 103–105 °C; ^{1}H NMR (500 MHz，CDCl$_3$) δ 7.78 (d, *J* = 8.0 Hz, 2H), 7.32-7.22 (m, 7H), 6.44 (d, *J* = 16.0 Hz, 1H), 6.02 (dt, *J* = 16.0, 6.5 Hz, 1H), 4.52 (t, *J* = 5.7 Hz, 1H), 3.77-3.75 (m, 2H), 2.42 (s, 3H).

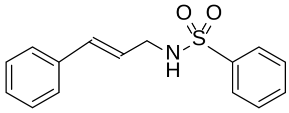

***N*-cinnamylbenzenesulfonamide** (**3b**) [2]: White solid; mp 69–70 °C; ^{1}H NMR (500 MHz，CDCl$_3$) δ 7.91-7.90 (m, 2H), 7.89-7.50 (m, 3H), 7.30-7.22 (m, 5H), 6.45 (d, *J* = 16.0 Hz, 1H), 6.01 (dt, *J* = 16.0, 6.0 Hz, 1H), 4.75 (t, *J* = 6.5 Hz, 1H), 3.79-3.77 (m, 2 H).

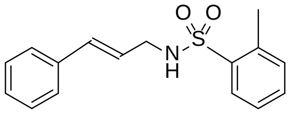

***N*-cinnamyl-2-methylbenzenesulfonamide** (**3c**): Pale yellow oil; ^{1}H NMR (500 MHz，CDCl$_3$) δ 8.00 (d, *J* = 8.0 Hz, 1H), 7.48-7.44 (m, 1H), 7.33-7.23 (m, 7H), 6.44 (d, *J* = 16.0 Hz, 1H), 6.01 (dt, *J* = 16.0, 6.5 Hz, 1H), 4.68 (t, *J* = 6.0 Hz, 1H), 3.75 (dd, *J* = 6.5, 6.0 Hz, 2H), 2.67 (s, 3H); ^{13}C NMR (125 MHz, CDCl$_3$) δ 138.0, 137.0, 136.0, 133.3, 132.9, 132.6, 129.6, 128.6, 128.0, 126.4, 126.3, 124.0, 45.4, 20.4; IR (KBr, cm^{-1}) 3452, 2994, 1769, 1758, 1637, 1384, 1318, 1245, 1158, 1131, 967, 758, 691, 594, 542; HRMS (ESI) calcd for $C_{16}H_{17}NO_2S$+Na 310.0878, found 310.0888.

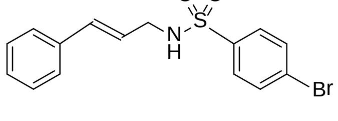

4-bromo-*N*-cinnamylbenzenesulfonamide (**3d**): Yellow solid; mp 53–55 °C; ^{1}H NMR (500 MHz，CDCl$_3$) δ 7.76-7.74 (m, 2H), 7.66-7.64 (m, 2H), 7.31-7.23 (m, 5H), 6.45 (d, *J* =

16.0 Hz, 1H), 6.01 (dt, J = 16.0, J = 6.5 Hz , 1H); 4.70 (t, J = 6.3 Hz, 1H), 3.79-3.77 (m, 2H); ^{13}C NMR (125 MHz, $CDCl_3$) δ 139.2, 135.8, 133.5, 132.4, 128.7, 128.6, 128.1, 127.8, 126.4, 123.6, 45.5; IR (KBr, cm^{-1}) 3421, 2984, 1654, 1637, 1560, 1508, 1383, 1241, 1162, 1096, 938, 848, 814, 668, 634, 608, 551; HRMS (ESI) calcd for $C_{15}H_{14}NO_2S$+Na 373.9826, found 373.9842.

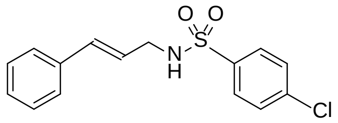

4-chloro-*N*-cinnamylbenzenesulfonamide (**3e**): White solid; mp 107–108 °C; ^{1}H NMR (500 MHz，$CDCl_3$) δ 7.82 (d, J = 8.5 Hz, 2H), 7.45 (d, J = 8.5 Hz, 2H), 7.30-7.22 (m, 5H), 6.44 (d, J = 16.0 Hz, 1H), 5.99 (dt, J = 16.0, 6.5 Hz, 1H), 4.90 (t, J = 6.0 Hz, 1H) , 3.78-3.75(m, 2H); ^{13}C NMR (125 MHz, $CDCl_3$) δ 139.2, 138.7, 135.9, 133.4, 129.4, 128.6, 128.1, 126.4, 123.7, 45.5; IR (KBr, cm^{-1}) 3444, 2993, 1633, 1477, 1383, 1245, 1162, 1094, 1050, 751, 693, 622; HRMS (ESI) calcd for $C_{15}H_{14}BrNO_2S$ 307.0428, found 307.0425.

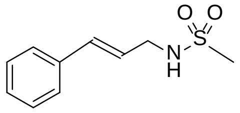

***N*-cinnamylmethanesulfonamide** (**3f**): White solid; mp 67–69 °C; ^{1}H NMR (500 MHz, $CDCl_3$) δ 7.38-7.35 (m, 2H), 7.34-7.30 (m, 2H), 7.28-7.25 (m, 1H), 6.61 (d, J = 16.0 Hz, 1H), 6.20 (dt, J = 16.0, 6.5 Hz, 1H), 4.82 (t, J = 5.5 Hz, 1 H), 3.93-3.90 (m, 2H), 2.98 (s, 3H); ^{13}C NMR (125 MHz, $CDCl_3$) δ 136.0, 133.3, 128.7, 128.1, 126.5, 124.5, 45.4, 41.1; IR (KBr, cm^{-1}) 3459, 2994, 1769, 1758, 1636, 1383, 1245, 1056, 913, 743, 626; HRMS (EI) calcd for $C_{10}H_{13}NO_2S$ 211.0667, found 211.0668.

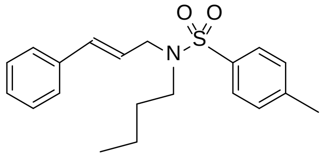

***N*-butyl-*N*-cinnamyl-4-methylbenzenesulfonamide** (**3g**): Yellow solid; mp 103–105 °C; ^{1}H NMR (500 MHz，CDCl$_3$) δ 7.72 (d, *J* = 8.5 Hz, 2H), 7.31-7.28 (m, 5H), 7.27-7.23 (q, *J* = 3.7 Hz, 2H), 6.44 (d, *J* = 15.5 Hz, 1H), 5.95 (dt, *J* = 15.5, 5.3 Hz, 1H), 3.96-3.94 (m, 2H), 3.16 (t, *J* = 7.5 Hz, 2H), 2.42 (s, 3H), 1.55-1.49(m, 2H), 1.31-1.23(m, 2H), 0.87 (t, *J* = 7.5 Hz, 3H); ^{13}C NMR (125 MHz, CDCl$_3$) δ 143.1, 137.3, 136.3, 133.5, 129.6, 128.7, 127.9, 127.2, 126.4, 124.4, 49.9, 47.1, 30.3, 21.5, 19.8, 13.7; IR (KBr, cm^{-1}) 3460, 3059, 2983, 2934, 1599, 1494, 1374, 1339, 1246, 1159, 1091, 912, 815, 738, 703, 653, 634, 608, 550; HRMS (EI) calcd for $C_{20}H_{25}NO_2S$ 343.1608, found 343.1612.

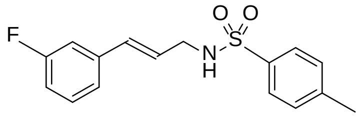

***N*-(3-fluorocinnamyl)-4-methylbenzenesulfonamide** (**3h**): Pale yellow solid; mp 81–82 °C; ^{1}H NMR (500 MHz，CDCl$_3$) δ 7.79-7.77 (m, 2H), 7.29-7.19 (m, 3H), 6.99-6.85 (m, 3H), 6.40 (d, *J* = 15.5 Hz, 1H), 6.00 (dt, *J* = 15.5, 6.5 Hz, 1H), 4.87 (t, *J* = 6.3 Hz, 1H) , 3.77-3.74(m, 2H), 2.41 (s, 3H); ^{13}C NMR (125 MHz, CDCl$_3$) δ 163.1 (d, *J* = 243.8 Hz), 143.8, 138.6 (d, *J* = 7.5 Hz), 137.1, 131.9, 130.2 (d, *J* = 8.8 Hz), 129.9, 127.3, 125.7, 122.5 (d, *J* = 2.5 Hz), 114.8 (d, *J* = 21.3 Hz), 112.9 (d, *J* = 22.5 Hz), 45.4, 21.6; IR (KBr, cm^{-1}) 3417，1636，1384，1246，1158，1092，550; HRMS(ESI) calcd for $C_{16}H_{16}FNO_2S$+Na 328.0783, found 328.0779.

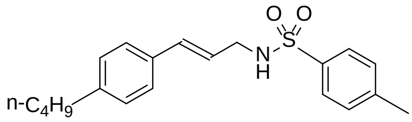

***N*-(4-butylcinnamyl)-4-methylbenzenesulfonamide** (**3i**): Pale yellow solid; mp 63–65 °C; ^{1}H NMR (500 MHz, $CDCl_3$) δ 7.77 (d, *J* = 8.0 Hz, 2H), 7.29 (d, *J* = 8.0 Hz, 2 H), 7.34 (d, *J* = 8.0 Hz, 2H), 7.09 (d, *J* = 8.0, 2H), 6.40 (d, *J* = 15.5 Hz, 1H), 5.95 (dt, *J* = 15.5, 6.5 Hz, 1H), 4.66 (t, *J* = 6.0 Hz, 1H), 3.74-3.71 (m, 2H), 2.57 (t, *J* = 7.5 Hz, 2H), 2.41 (s, 3H), 1.60-1.54 (m, 2H), 1.37-1.30(m, 2H), 0.91 (t, *J* = 7.5 Hz, 3H); ^{13}C NMR (125 MHz, $CDCl_3$) δ 143.5, 142.9, 137.1, 133.5, 133.1, 129.7, 128.6, 127.2, 126.3, 123.0, 45.6, 35.3, 33.5, 22.3, 21.5, 13.9; IR (KBr, cm^{-1}) 3412, 2930, 2858, 1638, 1618, 1512, 1384, 1331, 1245, 1161, 1094, 969, 913, 814, 734, 663, 607, 560, 551; HRMS (EI) calcd for $C_{20}H_{25}NO_2S$ 343.1606, found: 343.1618.

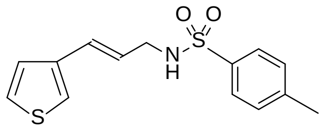

4-methyl-*N*-(3-(thiophen-3-yl)allyl)benzenesulfonamide (**3j**): Yellow solid; mp 97–100 °C; ^{1}H NMR (500 MHz，$CDCl_3$) δ 7.77 (d, *J* = 8.5, 2H), 7.31 (d, *J* = 8.0, 2H), 7.26-7.23(m, 1H), 7.08-7.06 (m, 2H), 6.46 (d, *J* = 16.0 Hz, 1H), 5.87 (dt, *J* = 16.0, 6.5 Hz, 1H), 4.53 (t, *J* = 6.3 Hz, 1H), 3.73-3.70 (m, 2H), 2.42 (s, 3H); ^{13}C NMR (125 MHz, $CDCl_3$) δ 143.6, 138.7, 137.1, 129.7, 127.2, 126.2, 124.8, 123.8, 122.7, 45.5, 29.7, 21.5; IR (KBr, cm^{-1}) 3414, 2923, 1617, 1493, 1447, 1384, 1331, 1265, 1164, 1091, 1071, 909, 813, 767, 739, 699, 674, 574, 548; HRMS (EI) calcd for $C_{14}H_{15}NO_2S_2$ 293.0544, found 293.0547.

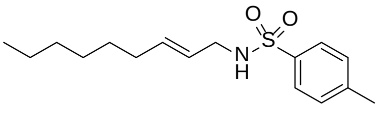

3ka

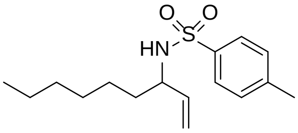

3kb

(*E*)-4-methyl-*N*-(non-2-enyl)benzenesulfonamide (**3ka**) and **4-methyl-*N*-(non-1-en-3-yl)benzenesulfonamide** (**3kb**): Pale yellow oil; ^{1}H NMR (500 MHz，CDCl$_3$) (**3ka**) δ 7.75 (d, *J* = 8.0 Hz, 2H), 7.30 (d, *J* = 8.0 Hz, 2H), 5.58-5.51(m, 1H), 5.32-5.27 (m, 1H), 4.66 (t, *J* = 6.0 Hz , 1H), 3.53-3.50 (m, 2H), 2.42 (s, 3H), 1.94-1.89 (m, 2H), 1.29-1.22 (m, 8H), 0.88-0.83(m, 3H); ^{1}H NMR (500 MHz，CDCl$_3$) (**3kb**) δ 7.76-7.73 (m, 2H), 7.28-7.26 (m, 2H), 5.58-5.51 (m, 1H), 5.01-4.93 (m, 2H), 4.82 (d, *J* = 7.5 Hz , 1H), 3.75-3.71 (m, 1H), 2.41 (s, 3H), 1.46-1.42 (m, 2H), 1.29-1.22 (m, 8H), 0.88-0.83(m, 3H); ^{13}C NMR (125 MHz, CDCl$_3$) (**3ka** and **3kb**) δ 143.3, 143.1, 138.2, 137.9, 137.2, 135.0, 129.6, 129.4, 127.2, 124.3, 115.7, 56.3, 45.3, 35.5, 32.0, 31.6, 31.6, 29.7, 28.8, 28.79, 28.7, 25.1, 22.5, 22.4, 21.4, 14.0, 13.9; IR (KBr, cm^{-1}) 3415, 2961, 2927, 2872, 1636, 1465, 1384, 1330, 1161, 1095, 1048, 970, 922, 814, 707, 666, 552; HRMS (EI) calcd for $C_{16}H_{25}NO_2S$ 295.1606, found 295.1607.

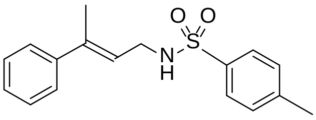

4-methyl-*N*-(3-phenylbut-2-enyl)benzenesulfonamide (**3l**): Yellow solid; mp 96–98 °C; ^{1}H NMR (500 MHz，CDCl$_3$) δ 7.78 (d, *J* = 8.5 Hz, 2H), 7.31-7.20 (m, 7H), 5.58-5.55 (m, 1H), 4.65 (t, *J* = 5.8 Hz, 1H), 3.78 (t, *J* = 6.5 Hz, 2H), 2.41 (s, 3H), 1.95 (s, 3H); ^{13}C NMR (125 MHz, CDCl$_3$) δ 143.5, 142.4, 139.3, 137.1, 129.7, 128.2, 127.4, 127.2, 125.7, 121.9, 41.5, 21.5, 16.0; IR (KBr, cm^{-1}) 3414, 2959, 2926, 1715, 1617, 1465, 1384, 1328,

1160, 1093, 813, 761, 700, 662, 550; HRMS (EI) calcd for $C_{17}H_{19}NO_2S$ 301.1126, found 301.1122.

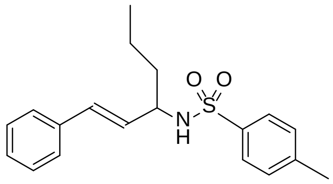

(*E*)-4-methyl-*N*-(1-phenylhex-1-en-3-yl)benzenesulfonamide (**3m**): White solid; mp 75–77 °C; ^{1}H NMR (500 MHz，CDCl$_3$) δ 7.74-7.72 (m, 2H), 7.25-7.14 (m, 5H), 7.09-7.07 (m, 2H), 6.17 (d, *J* = 15.5 Hz, 1H), 5.69 (dd, *J* = 15.5 Hz, *J* = 7.5 Hz, 1H), 5.12 (d, *J* = 8.0 Hz, 1H), 3.94-3.88 (m, 1H) , 2.27 (s, 3H), 1.57-1.47 (m, 2H), 1.35-1.24 (m, 2H), 0.85 (t, *J* = 7.5 Hz, 3H); ^{13}C NMR (125 MHz, CDCl$_3$) δ 143.1, 138.2, 136.3, 131.3, 129.4, 129.0, 128.3, 127.5, 127.3, 126.3, 56.2, 38.0, 21.3, 18.6, 13.6; IR (KBr, cm^{-1}) 3275, 3058, 2958, 2931, 2871, 1598, 1448, 1384, 1324, 1159, 1093, 1028, 966, 813, 746, 667, 573; HRMS (EI) calcd for $C_{19}H_{23}NO_2S$ 329.1446, found 329.1451.

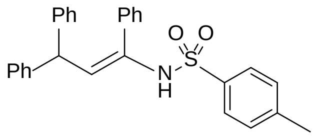

4-methyl-*N*-(1,3,3-triphenylprop-1-enyl)benzenesulfonamide (**3n**): Yellow solid; mp 113–114 °C; ^{1}H NMR (500 MHz，CDCl$_3$) δ 7.60 (d, *J* = 7.5 Hz, 2H), 7.46-7.45 (m, 2H), 7.45-7.20 (m, 11H), 6.94-6.92 (m, 4H), 6.06 (s, 1H), 5.93 (d, *J* = 9.0 Hz, 1H), 4.38 (d, *J* = 9.0 Hz, 1H) , 2.42 (s, 3H); ^{13}C NMR (125 MHz, CDCl$_3$) δ 143.8, 142.8, 137.2, 136.8, 135.4, 129.6, 128.8, 128.6, 128.0, 127.9, 127.5, 127.3, 126.8, 48.7, 21.5; IR (KBr, cm^{-1}) 3418, 2924, 2853, 1617, 1456, 1384, 1326, 1265, 1160, 1093, 965, 814, 742, 705, 664, 551; HRMS (EI) calcd for $C_{28}H_{25}NO_2S$ 439.1606, found 439.1610.

References

[1] Carta, F.; Pothen, B.; Maresca, A. *Chem. Biol. Drug Des.* **2009,** *74,* 196. doi:10.1111/j.1747-0285.2009.00842.x

[2] Gensler, W. J.; Dheer, S. K. *J. Org. Chem.* **1981,** *46,* 4051. doi:10.1021/jo00333a023

chj081107 CDCl3

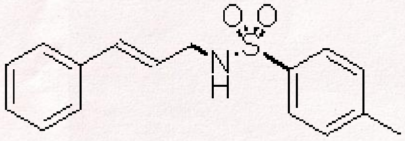

3a

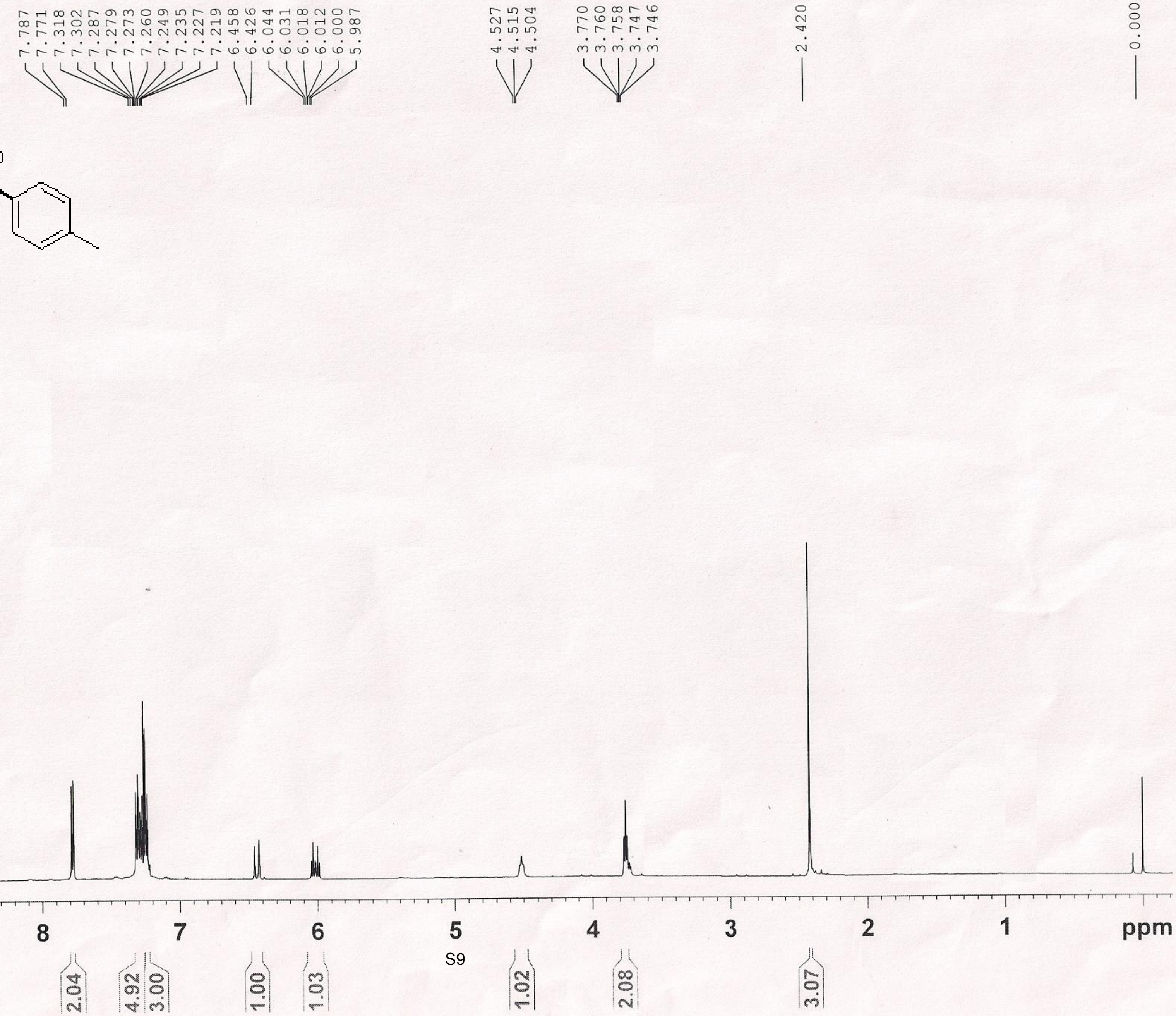

chj081225 CDCl3

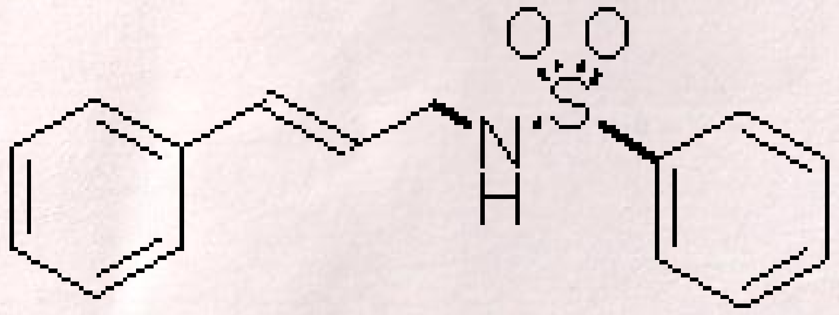

3b

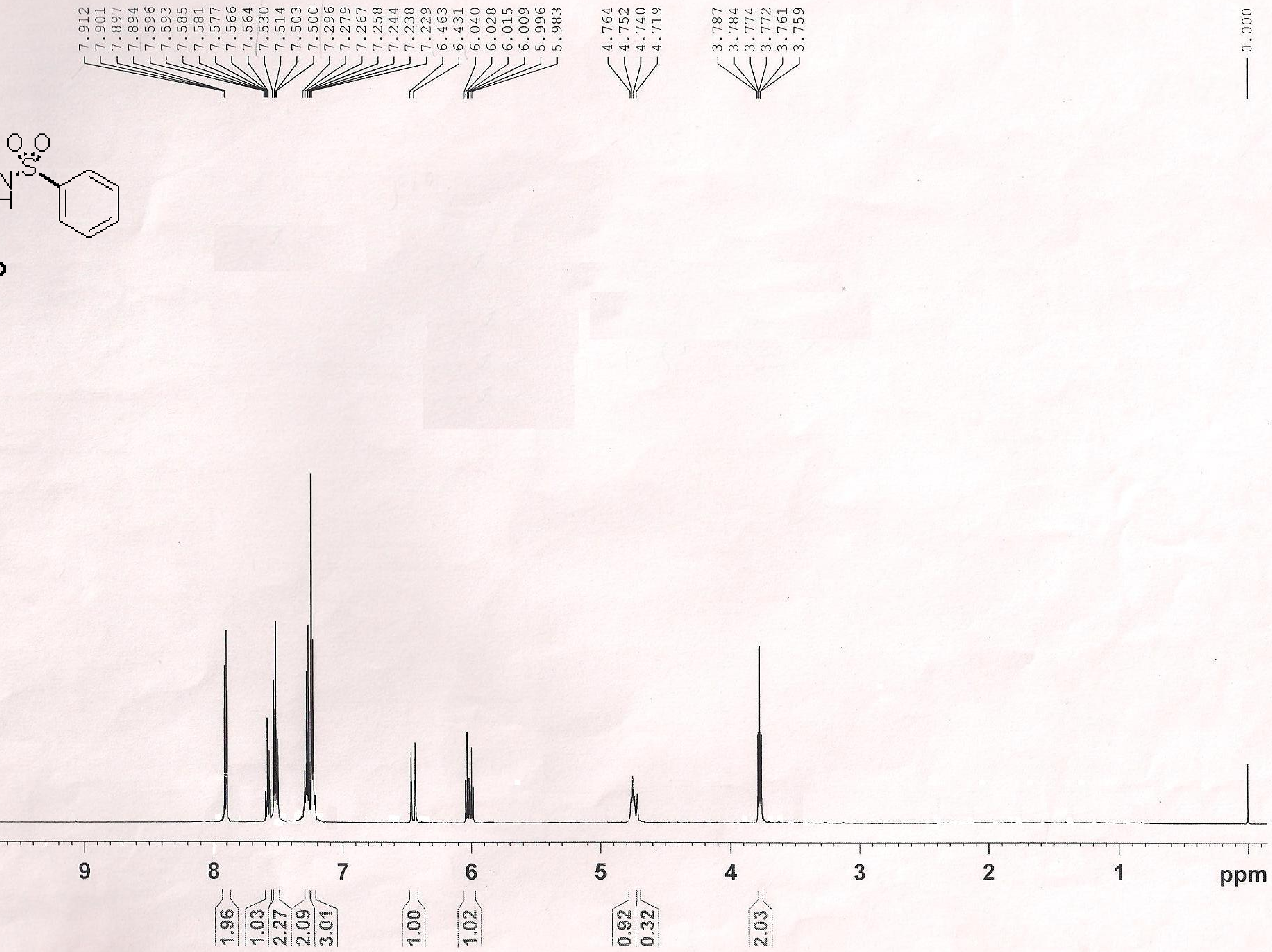

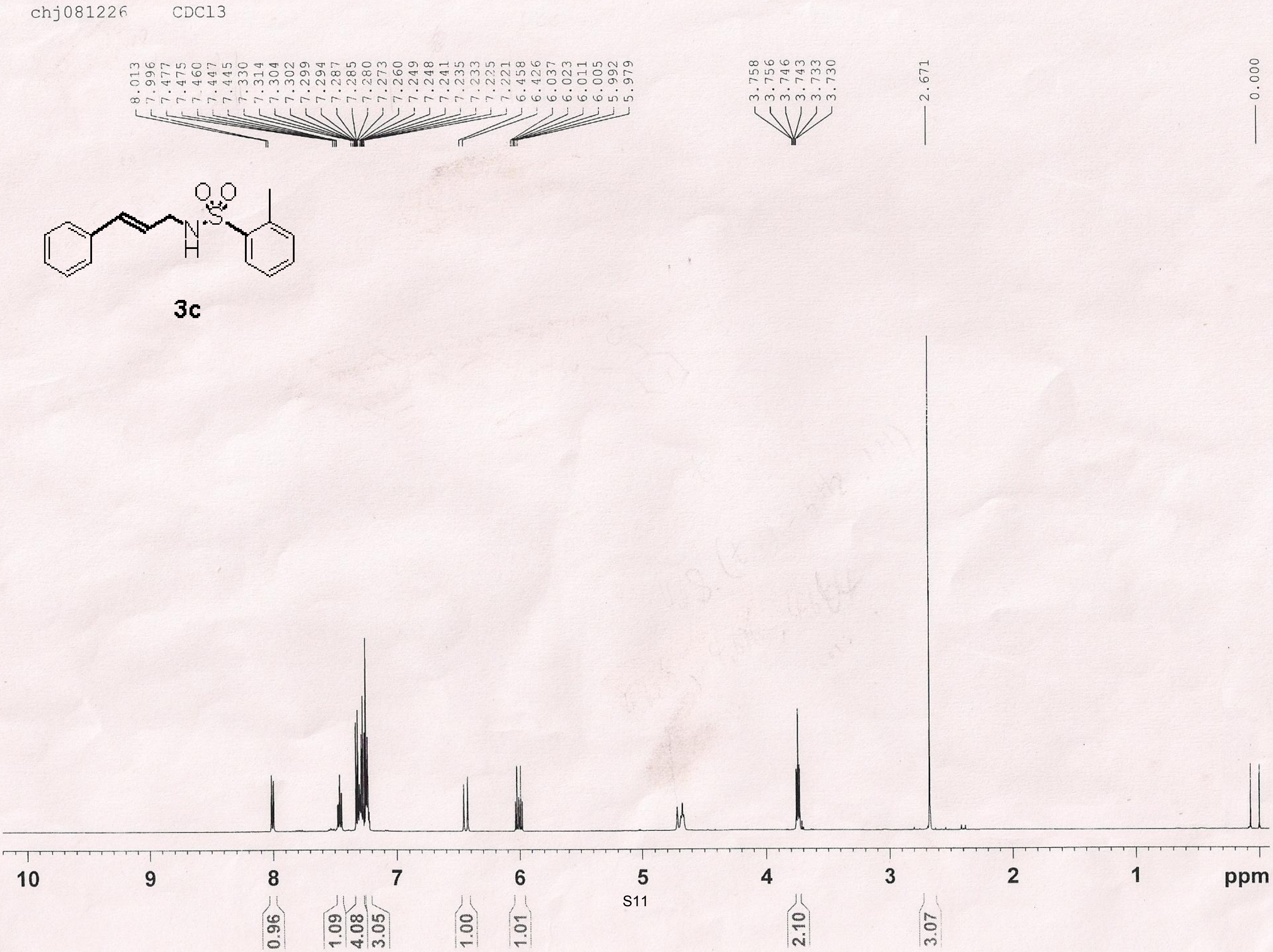
chj081226 CDCl3
8.013
7.996
7.477
7.475
7.460
7.447
7.445
7.330
7.314
7.304
7.302
7.299
7.294
7.287
7.285
7.280
7.273
7.260
7.249
7.248
7.241
7.235
7.233
7.225
7.221
6.458
6.426
6.037
6.023
6.011
6.005
5.992
5.979
3.758
3.756
3.746
3.743
3.733
3.730
2.671
0.000
3c
10
9
8
7
6
5
4
3
2
1
ppm
0.96
1.09
4.08
3.05
1.00
1.01
2.10
3.07

chj081226 CDCl3

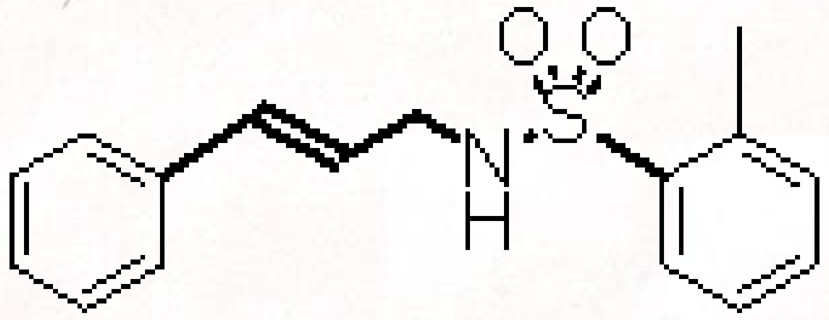

3c

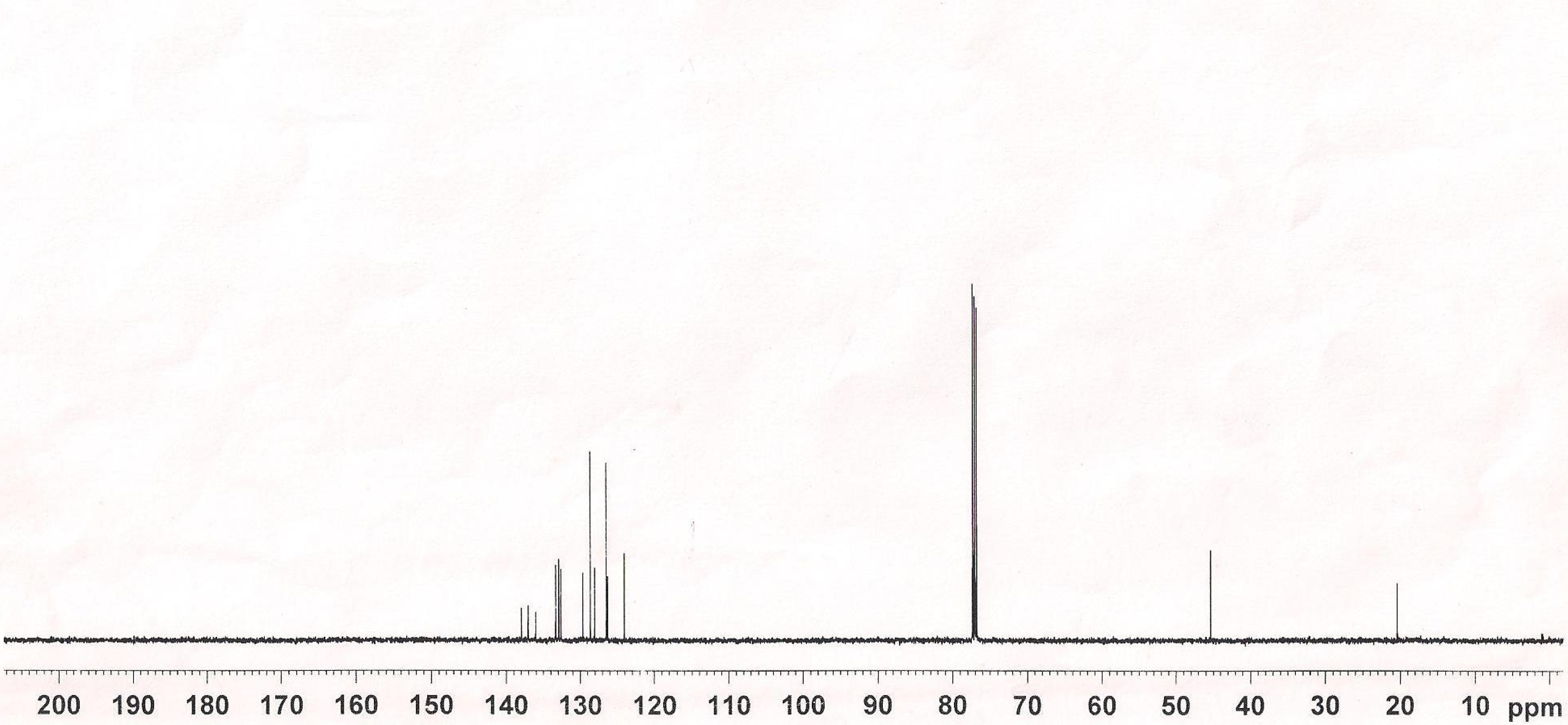

chj09105 CDCl3

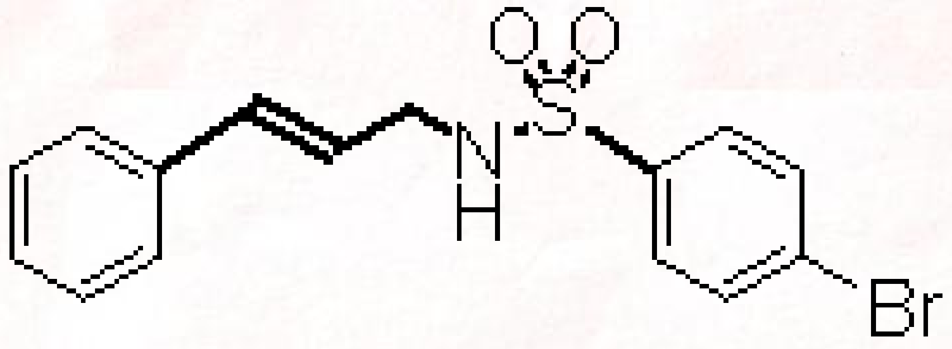

3d

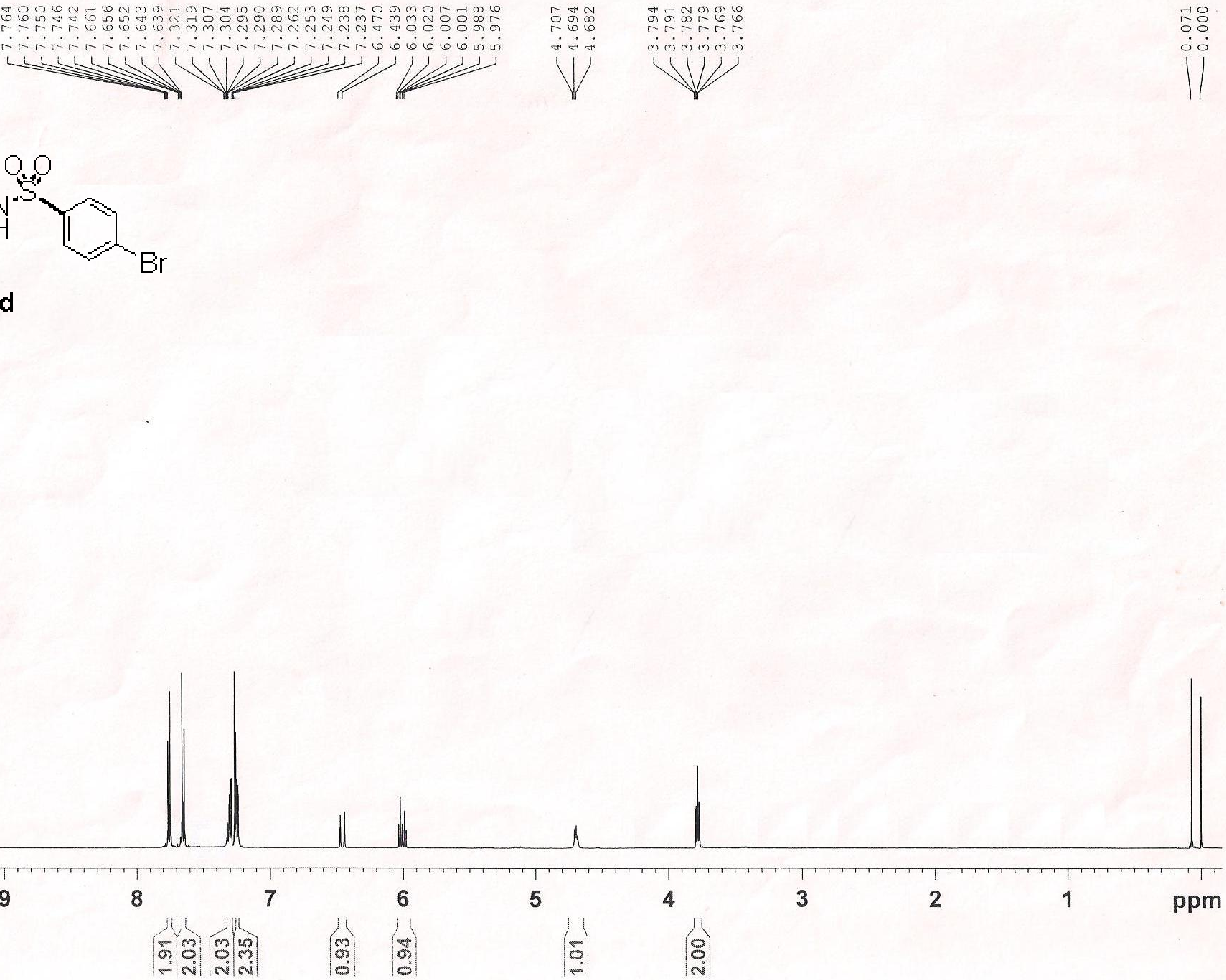

chj090105 CDCl3

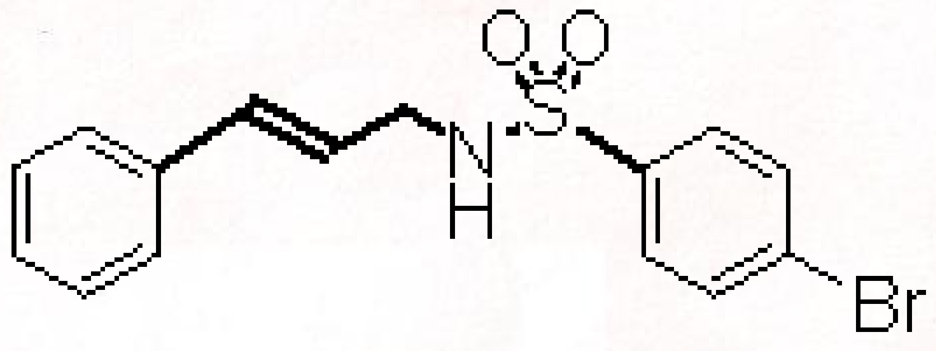

3d

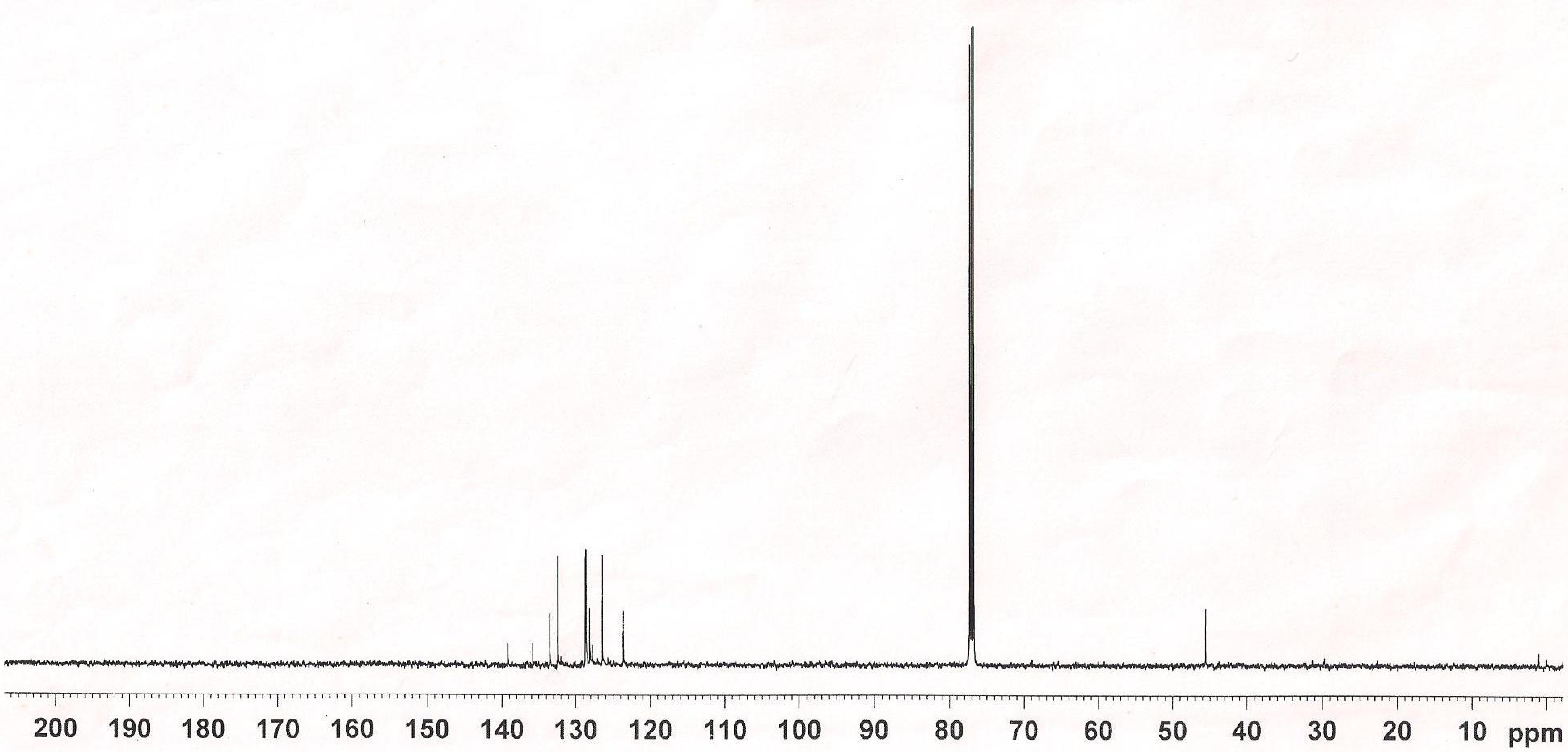

CHJ091127 CDCl3

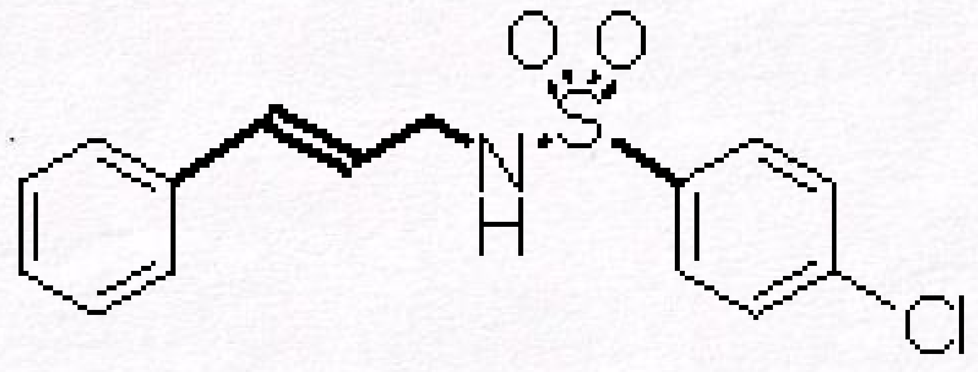

3e

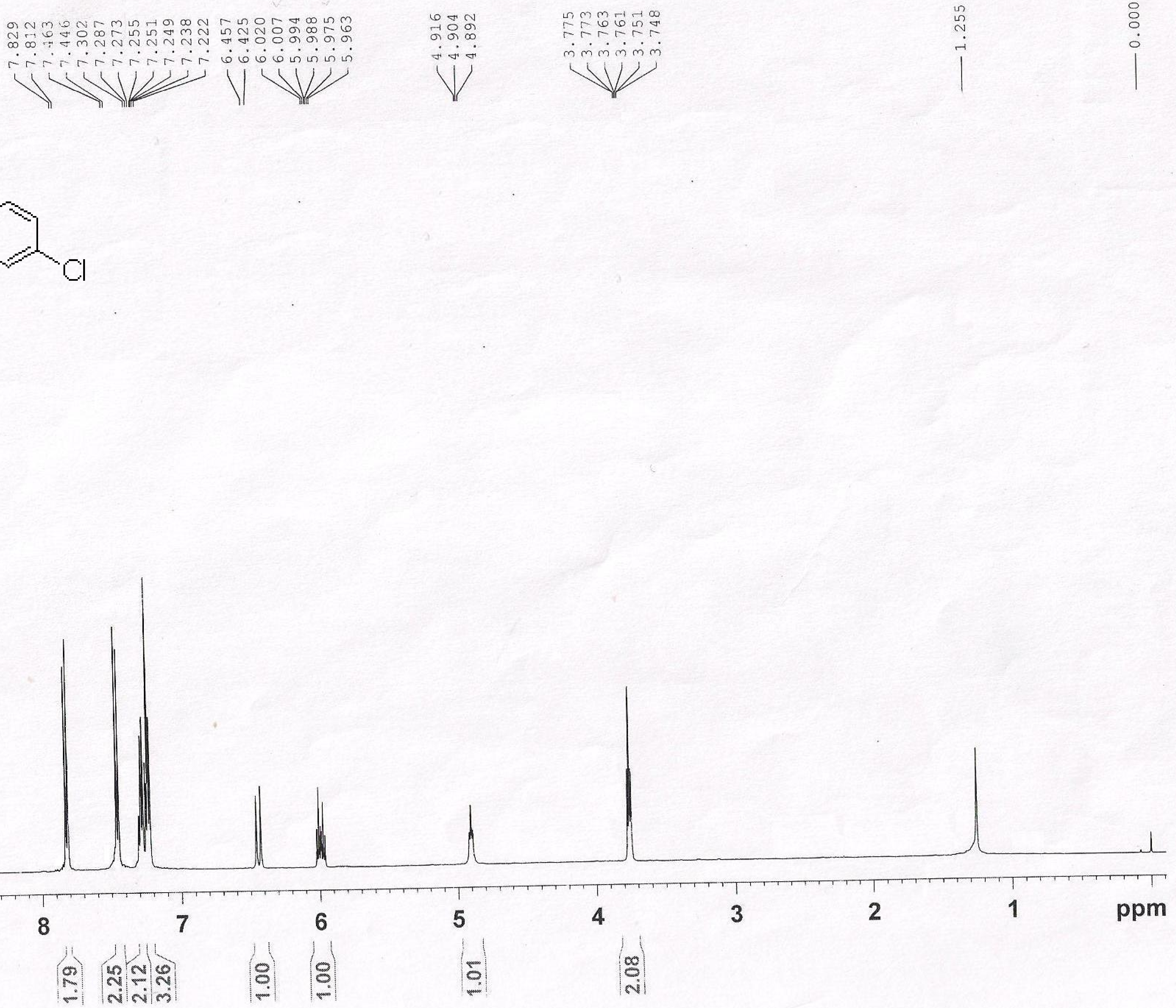

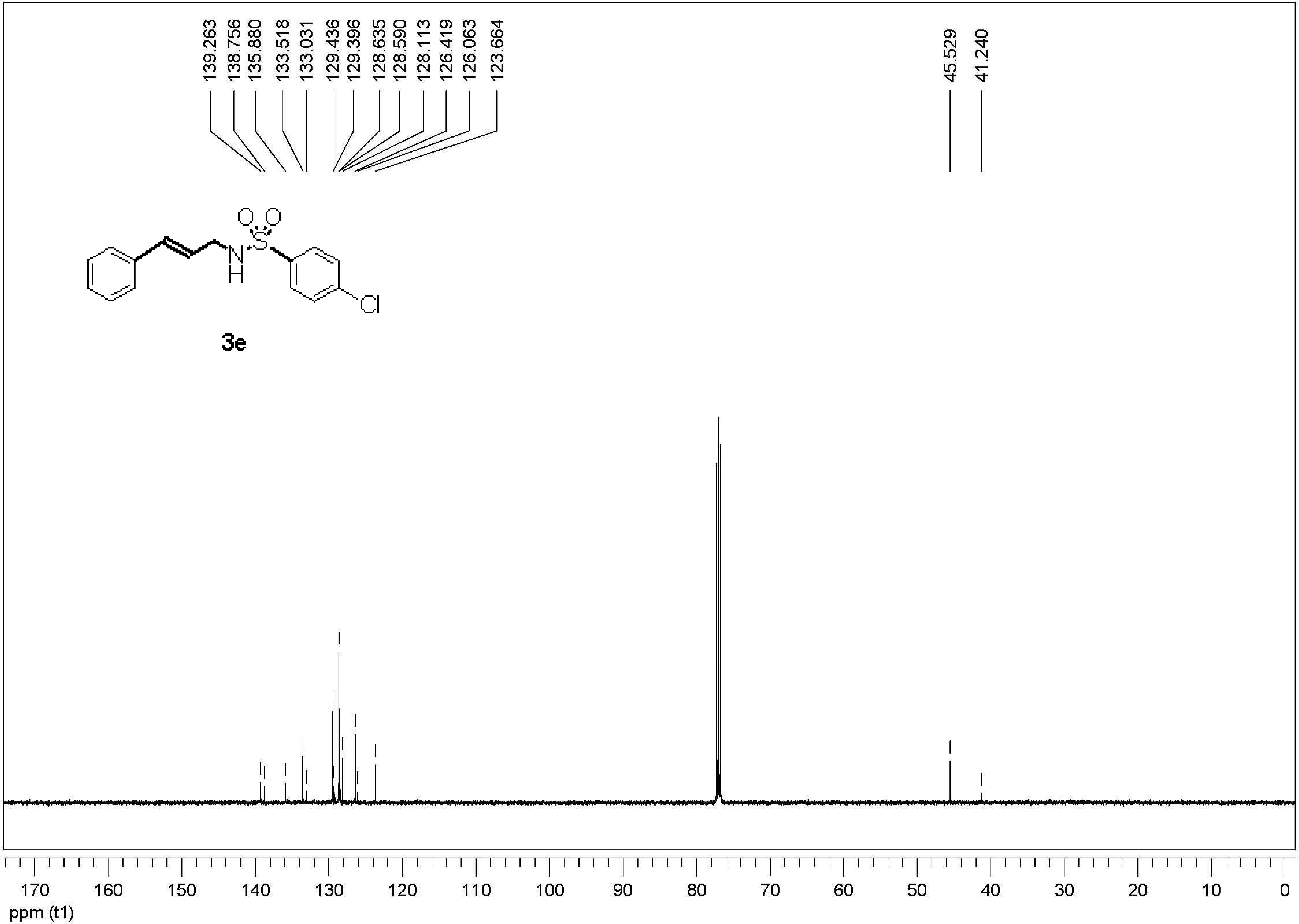
139.263
138.756
135.880
133.518
133.031
129.436
129.396
128.635
128.590
128.113
126.419
126.063
123.664
45.529
41.240
3e
170
160
150
140
130
120
110
100
90
80
70
60
50
40
30
20
10
0
ppm (t1)

chj081229   CDCl3

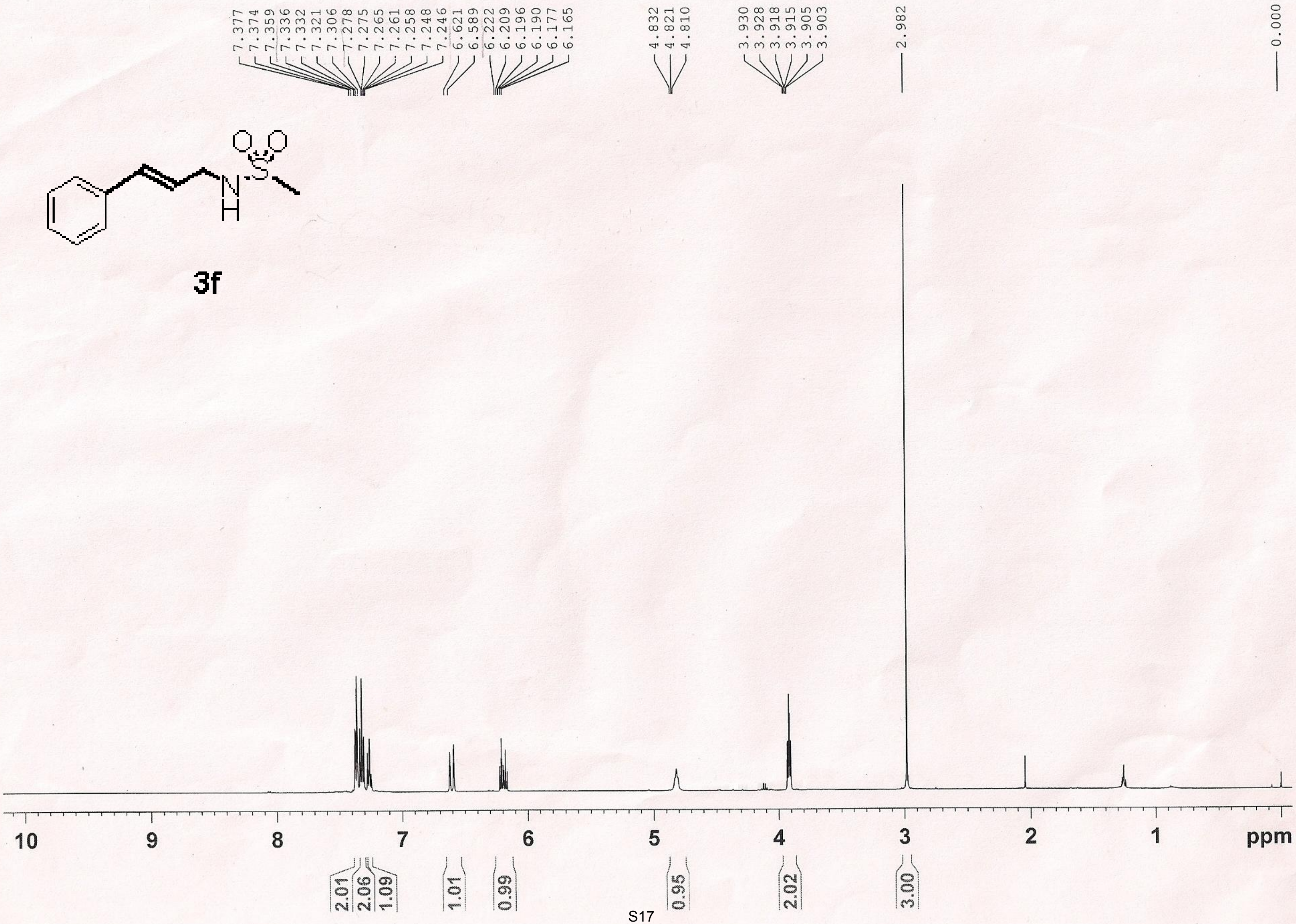

CHJ081229 CDCl3

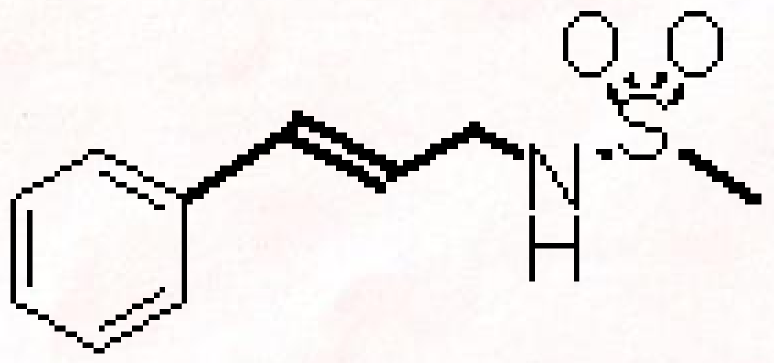

3f

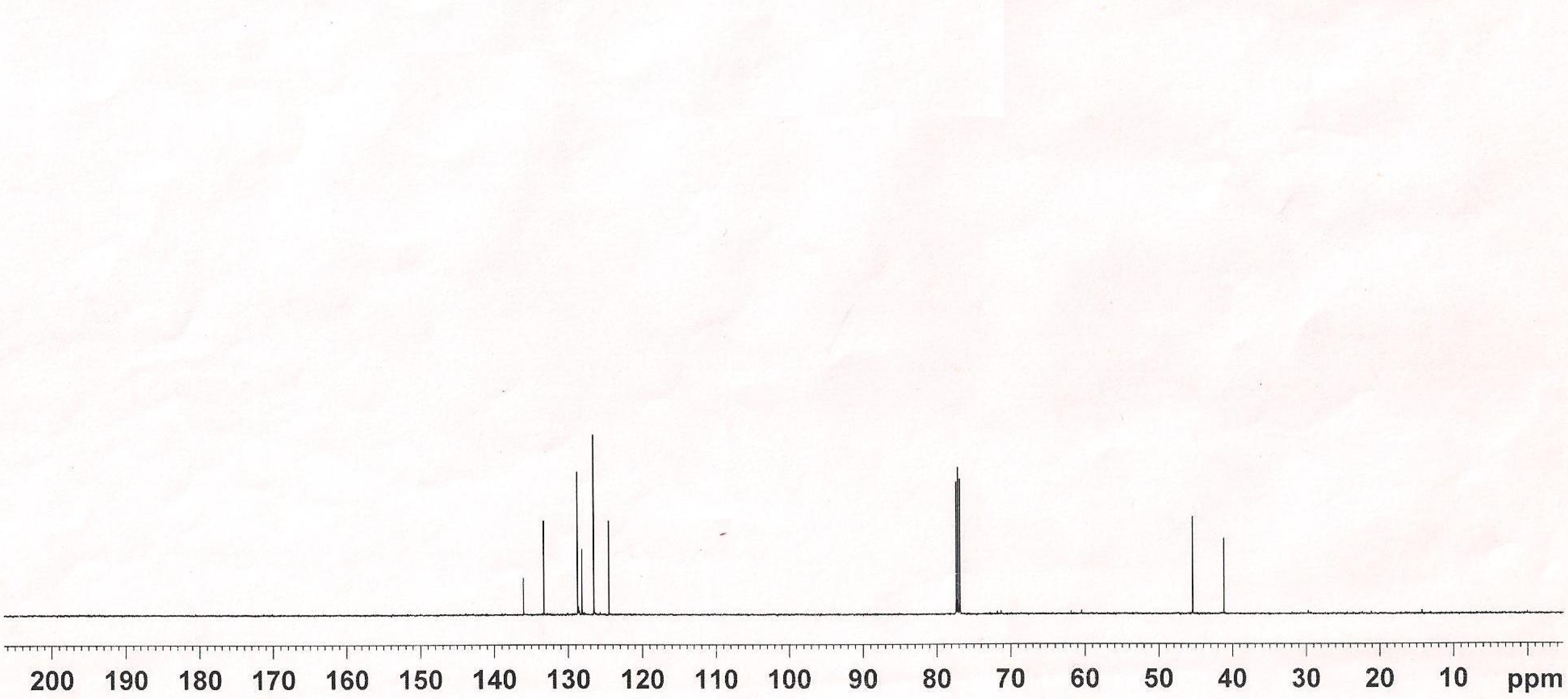

CHJ100408 CDCl3

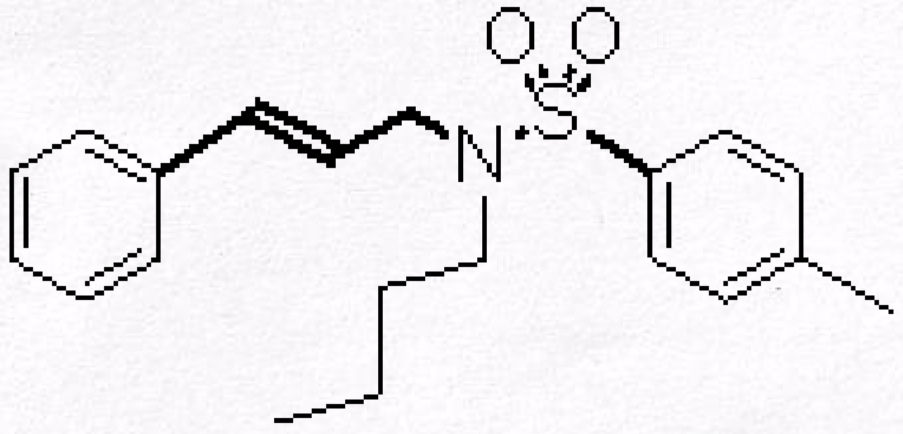

3g

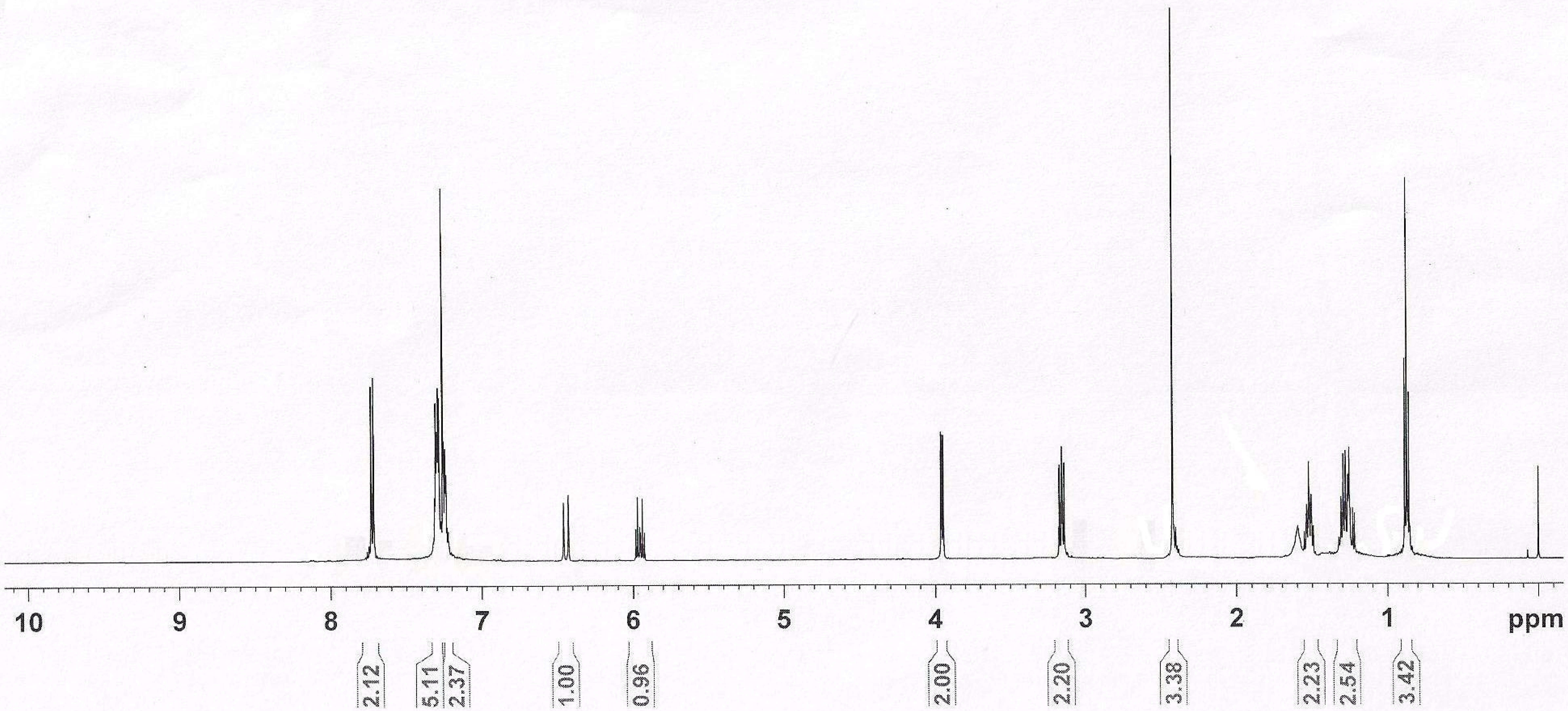

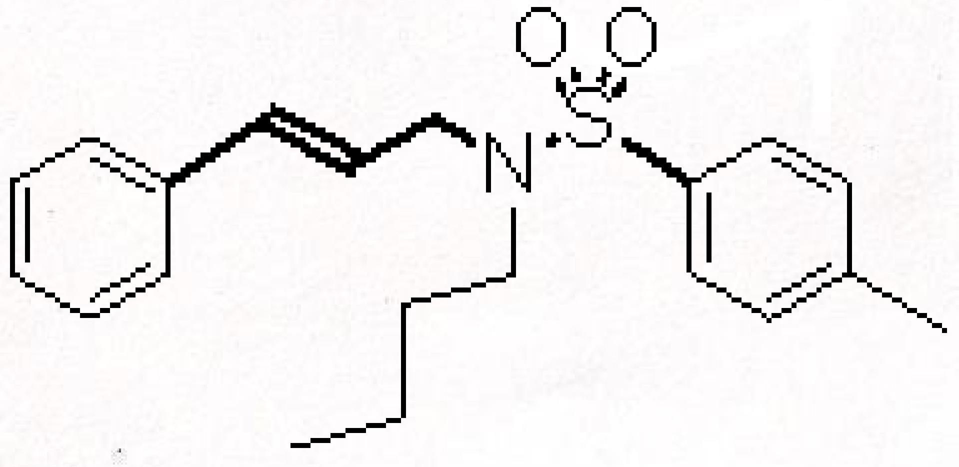

3g

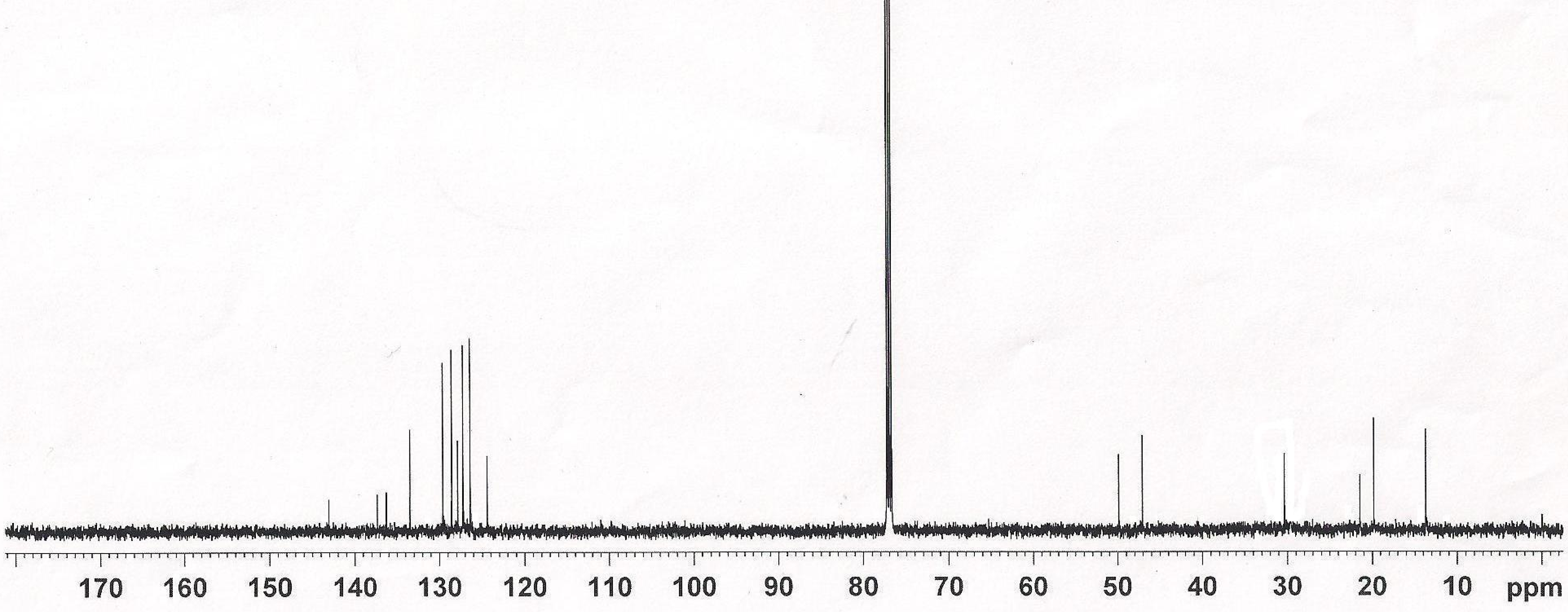

chj100129c CDCl3
143.08
137.34
136.27
133.46
129.62
128.56
127.85
127.21
126.39
124.43
77.25
77.00
76.75
49.89
47.10
30.33
21.46
19.81
13.67
170
160
150
140
130
120
110
100
90
80
70
60
50
40
30
20
10
ppm

CHJ090310 CDCl3

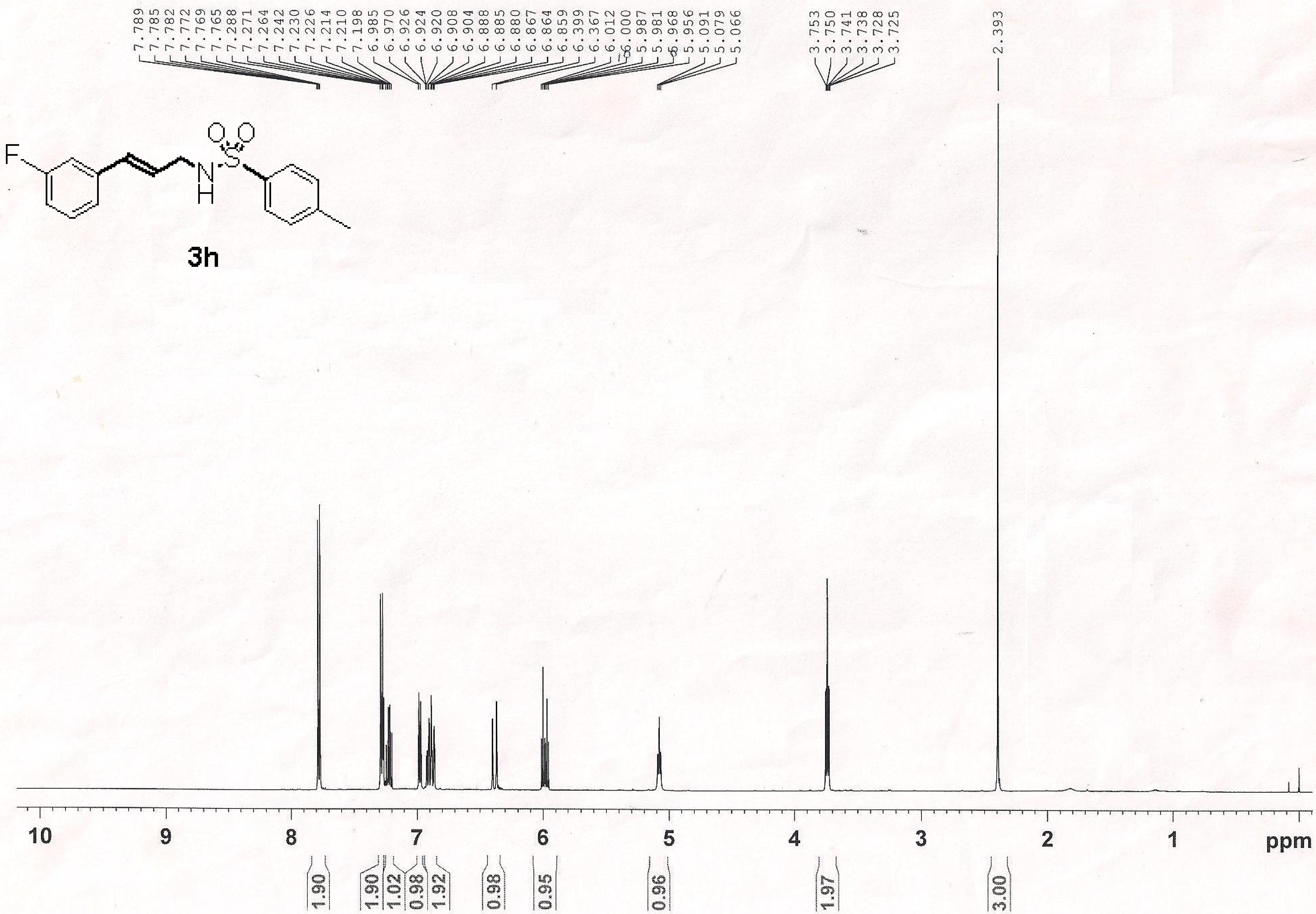

chj081222 CDCl3

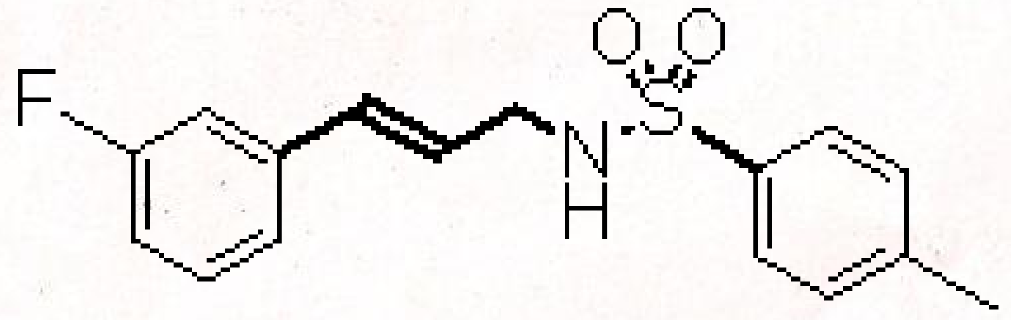

3h

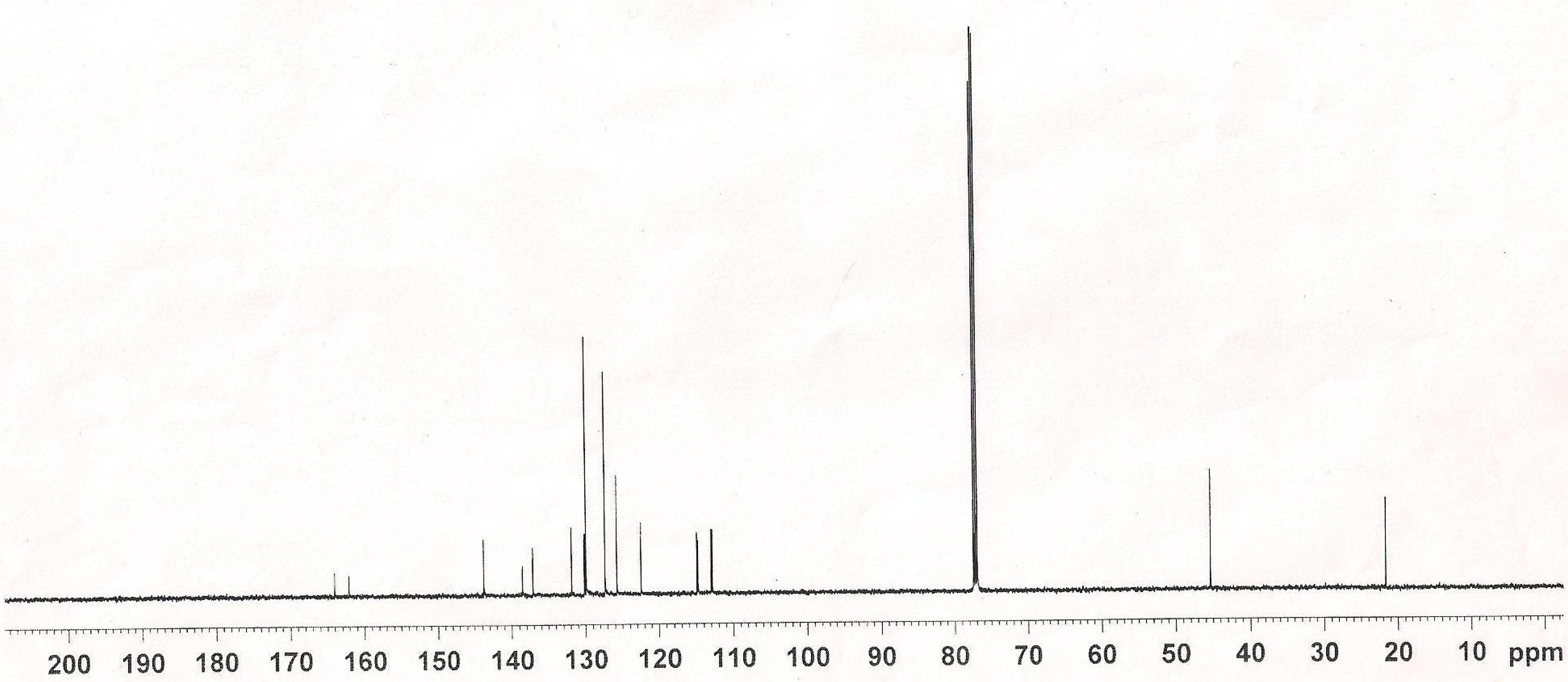

chjdingji CDCl3

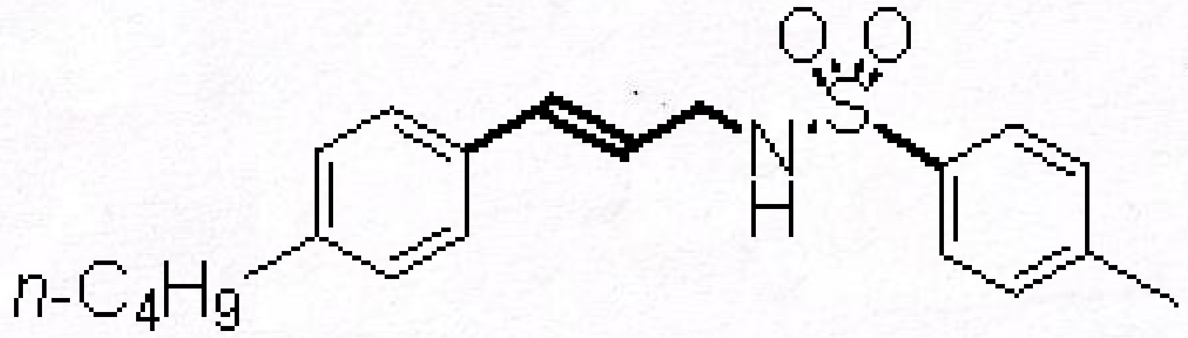

3i

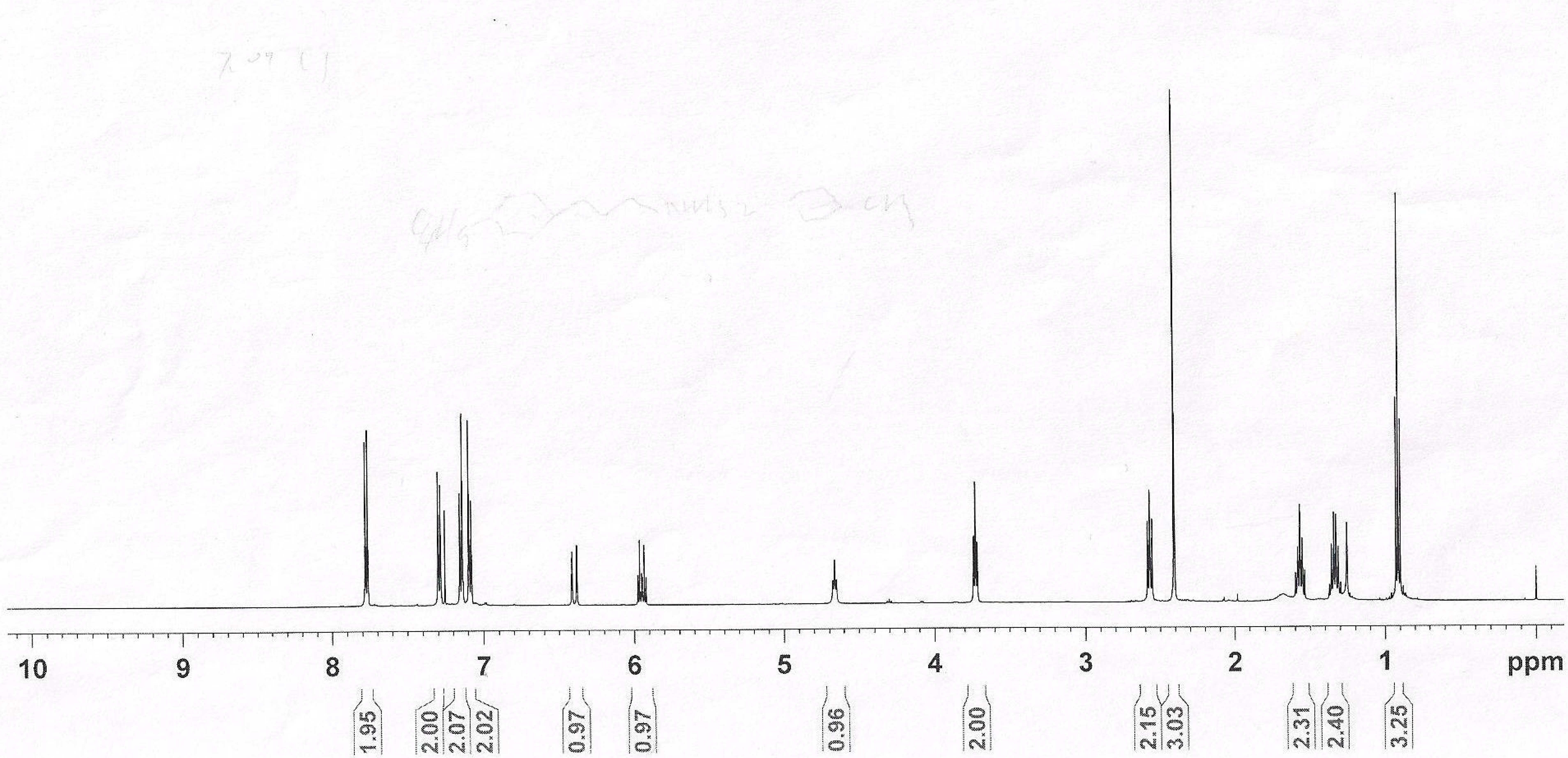

chj090210 CDCl3

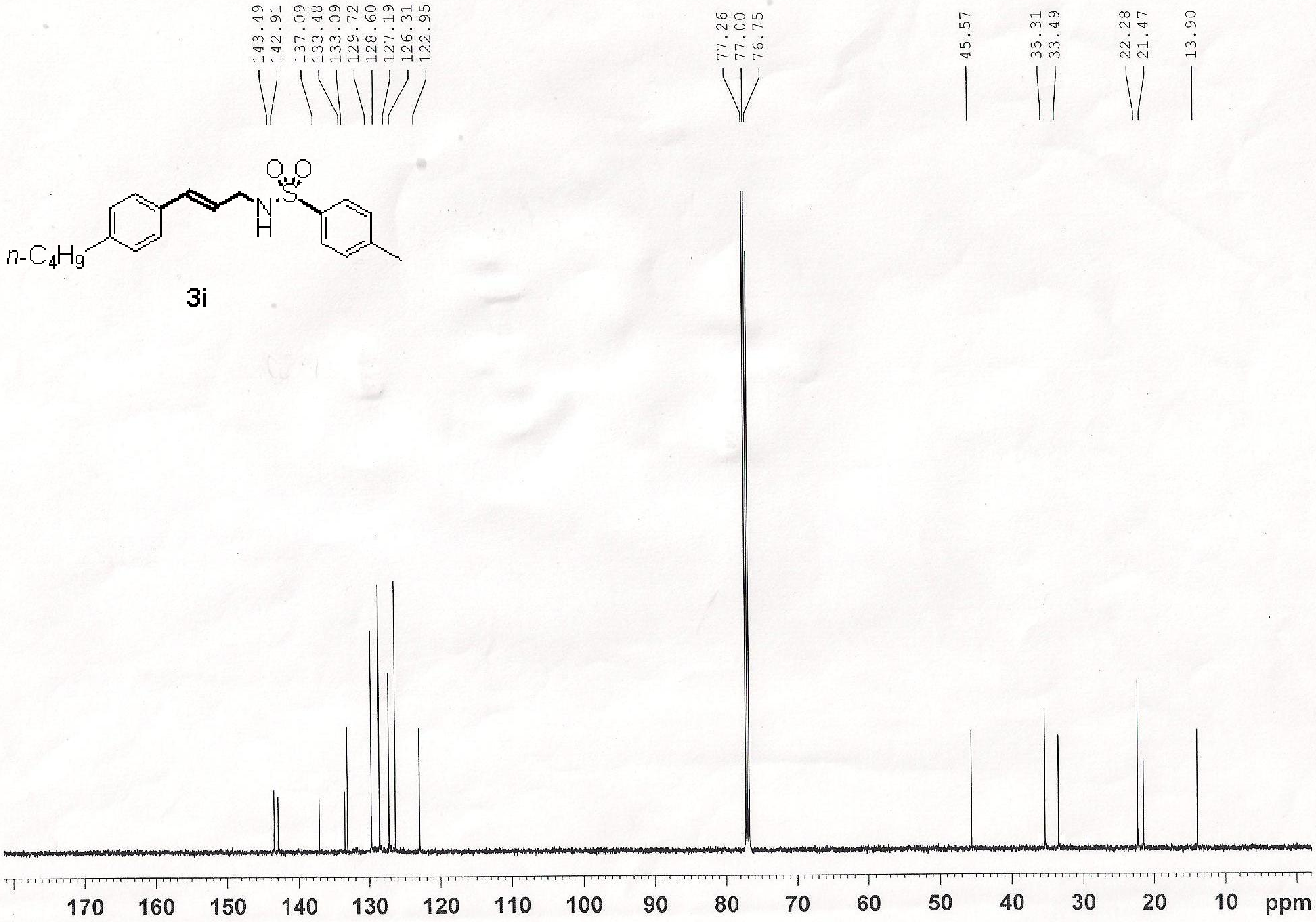

chj100201 CDCl3

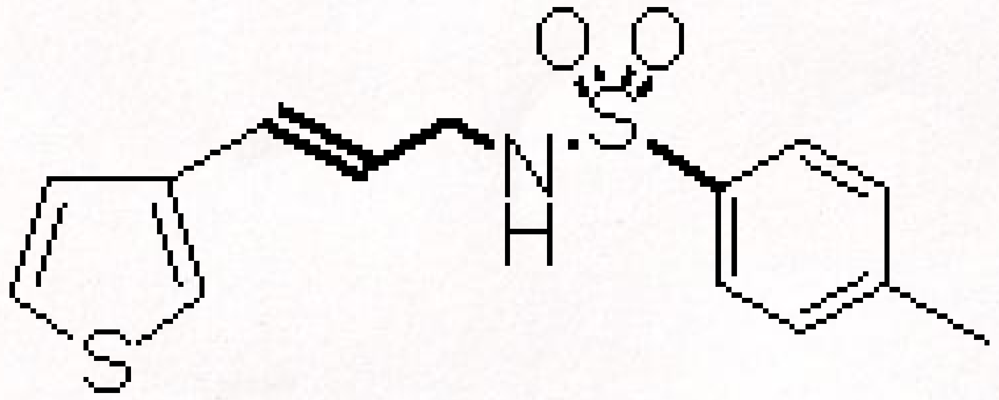

3j

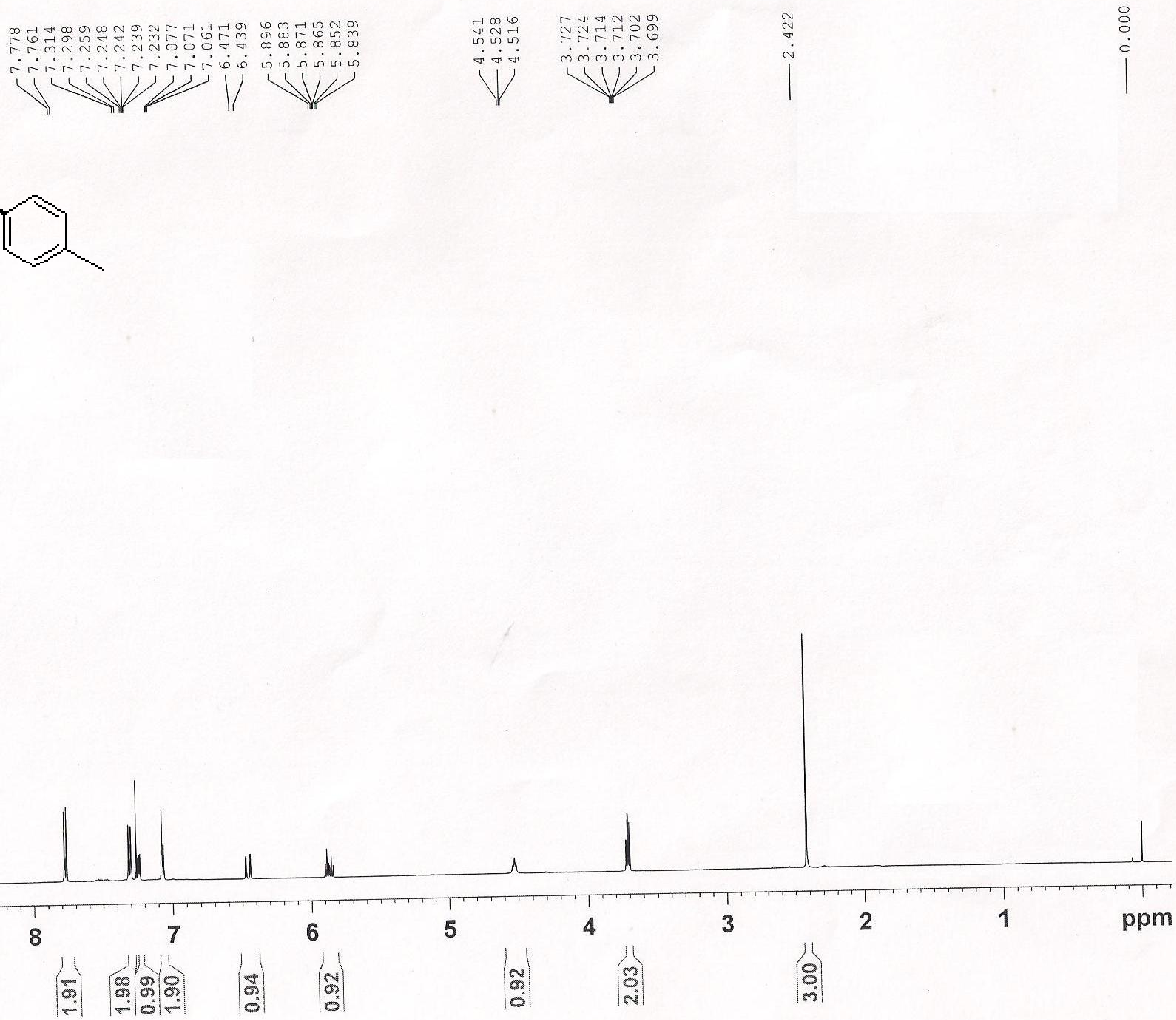

CHJ100308 CDCl3

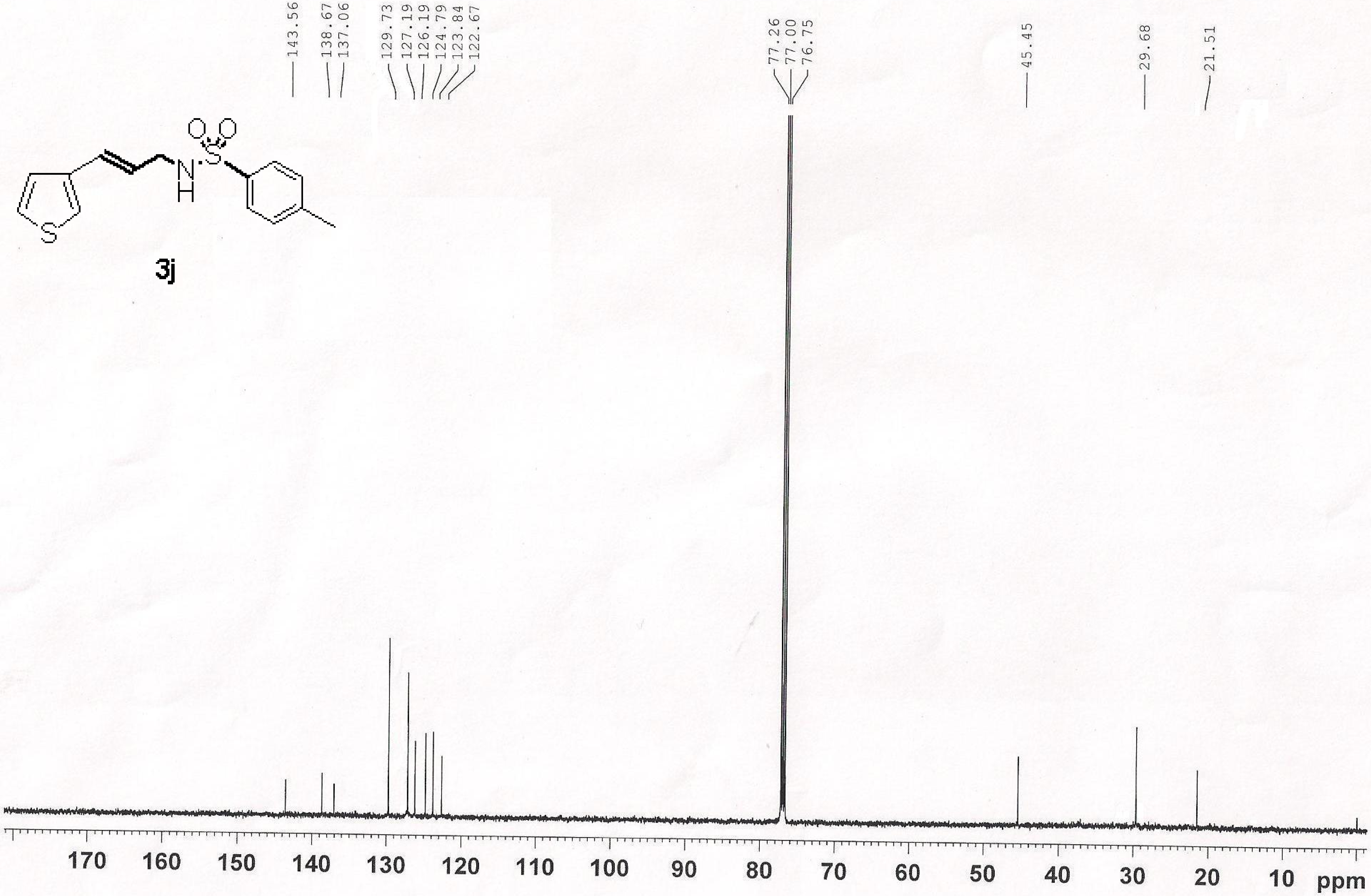

chj090608 CDCl3

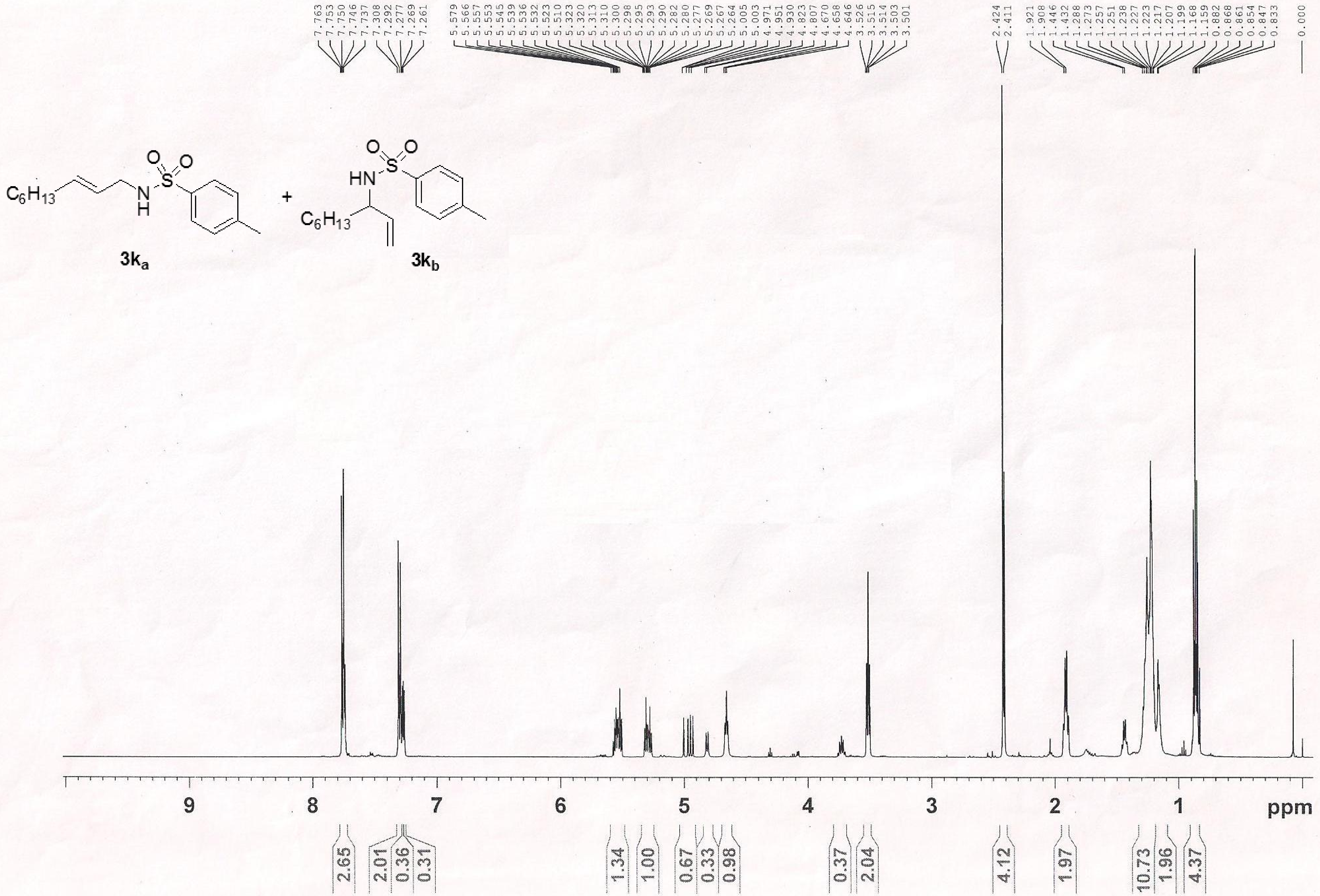

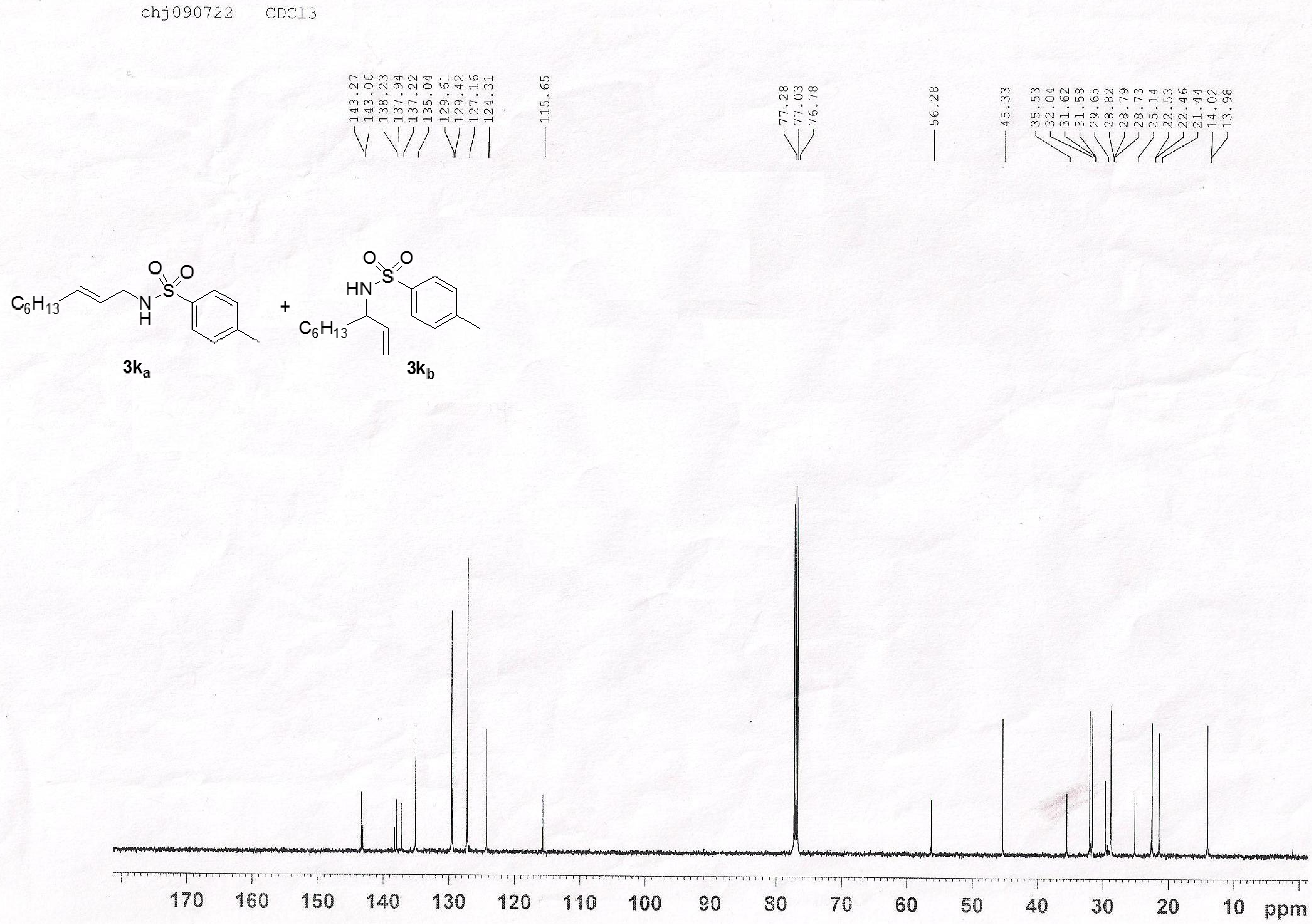
chj090722 CDCl3
143.27
143.06
138.23
137.94
137.22
135.04
129.61
129.42
127.16
124.31
115.65
77.28
77.03
76.78
56.28
45.33
35.53
32.04
31.62
31.58
29.65
28.82
28.79
28.73
25.14
22.53
22.46
21.44
14.02
13.98
C_6H_{13}
3k$_a$
+
C_6H_{13}
3k$_b$
170 160 150 140 130 120 110 100 90 80 70 60 50 40 30 20 10 ppm

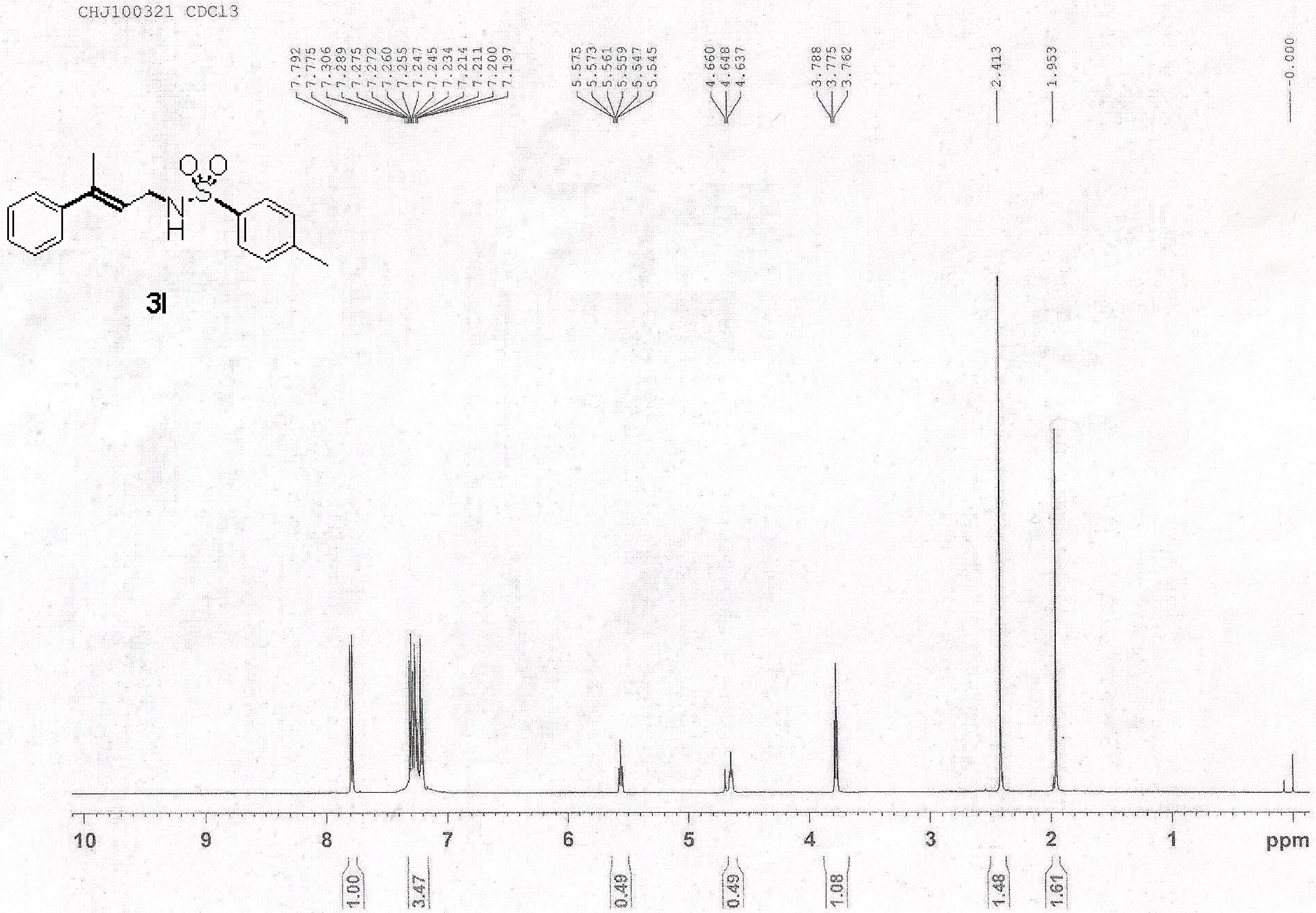
CHJ100321 CDCl3
7.792
7.775
7.306
7.289
7.275
7.272
7.260
7.255
7.247
7.245
7.234
7.214
7.211
7.200
7.197
5.575
5.573
5.561
5.559
5.547
5.545
4.660
4.648
4.637
3.788
3.775
3.762
2.413
1.953
-0.000
3l
10
9
8
7
6
5
4
3
2
1
ppm
1.00
3.47
0.49
0.49
1.08
1.48
1.61

chj100321C CDCl3

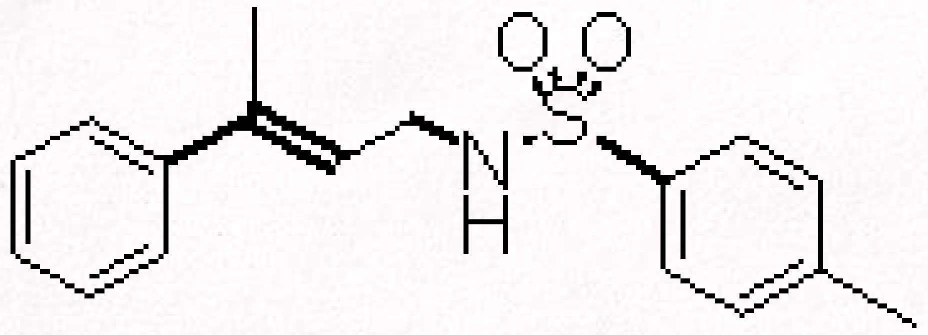

3l

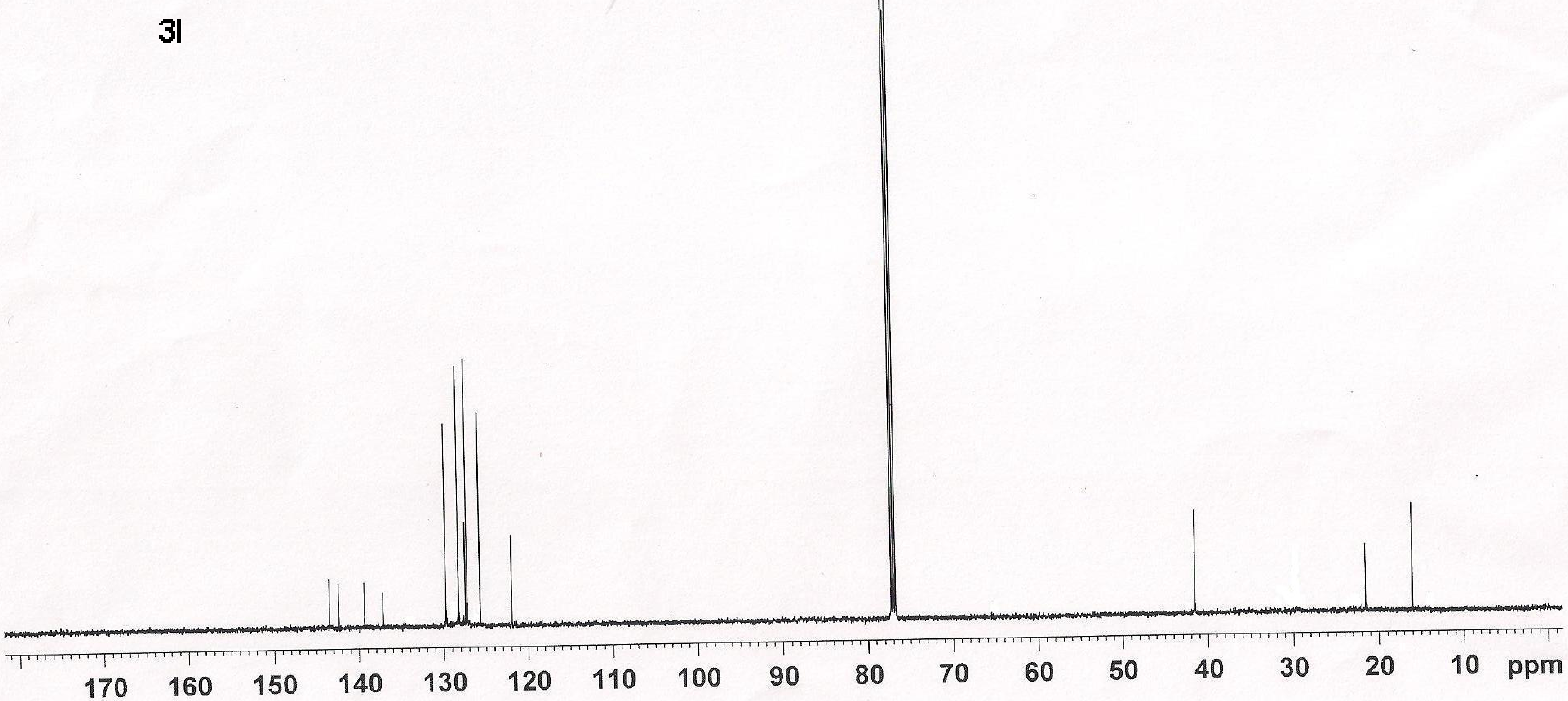

chjneibu CDCl3

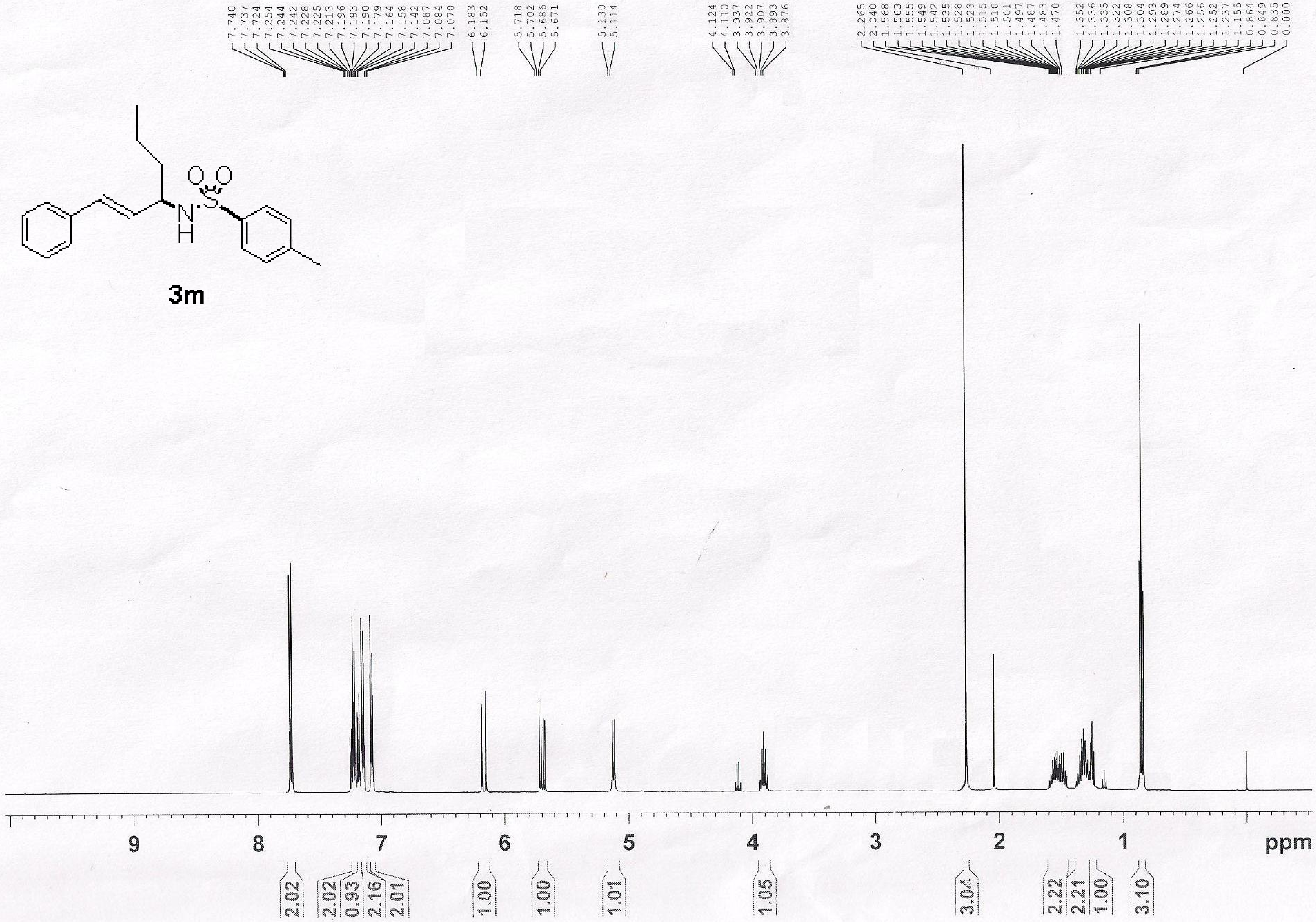

3m

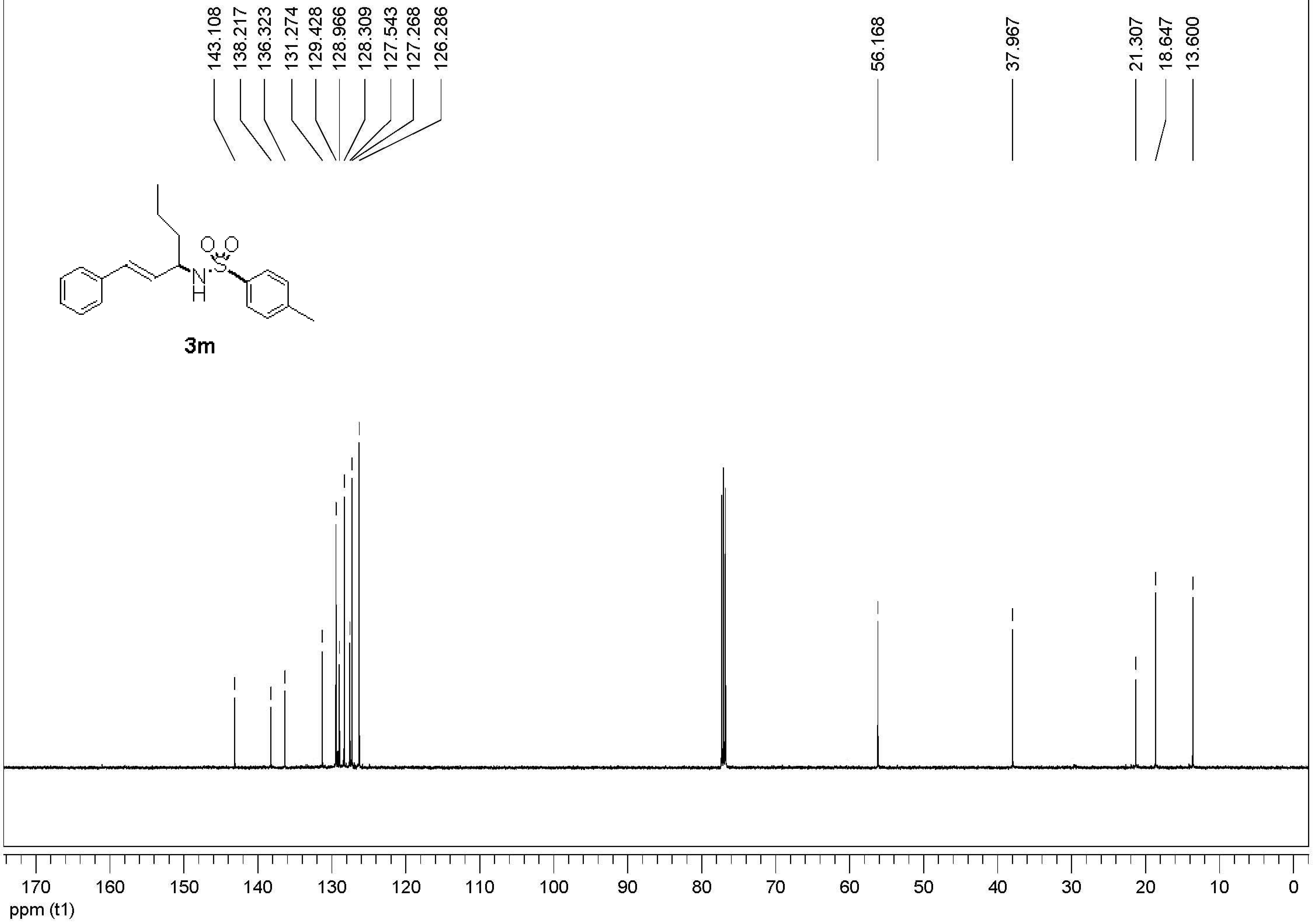
143.108
138.217
136.323
131.274
129.428
128.966
128.309
127.543
127.268
126.286
56.168
37.967
21.307
18.647
13.600
3m
170
160
150
140
130
120
110
100
90
80
70
60
50
40
30
20
10
0
ppm (t1)

CHJ100323 CDCl3

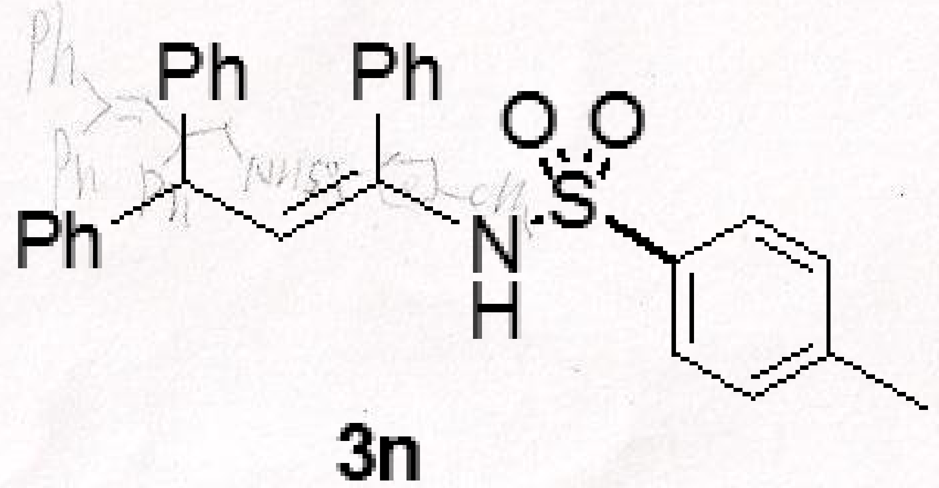

3n

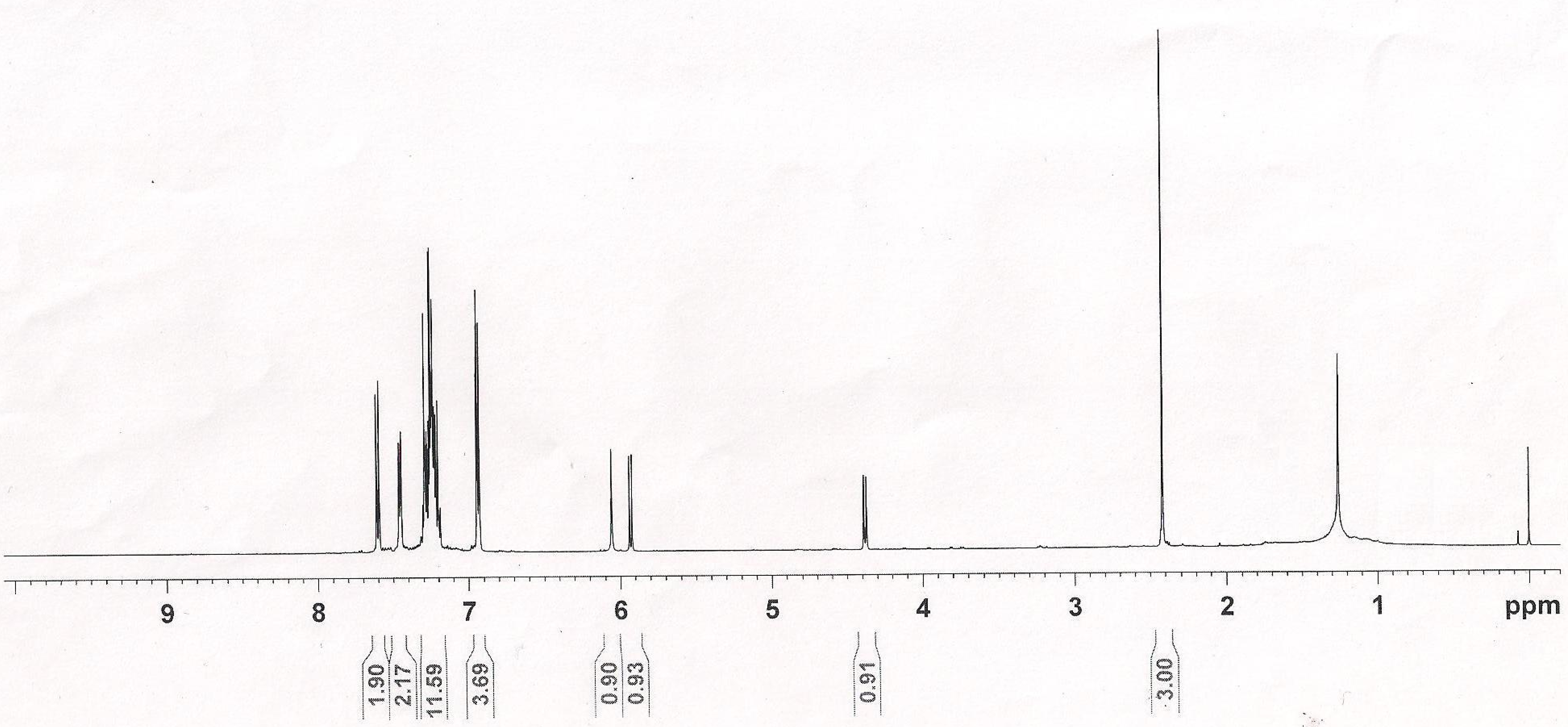

chj100324 CDCl3

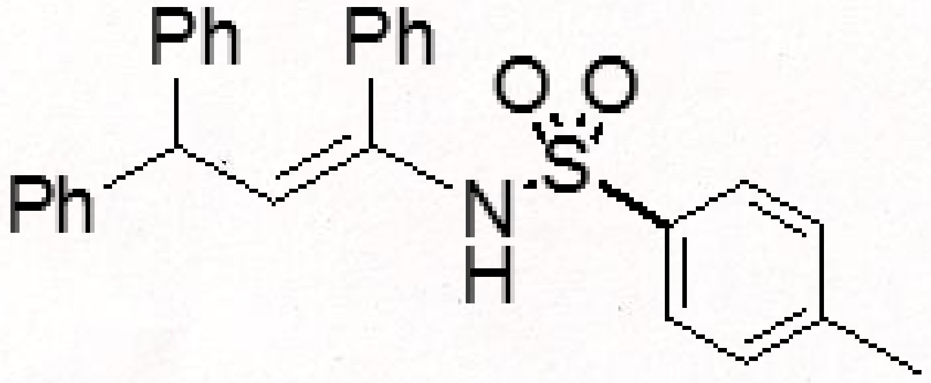

3n

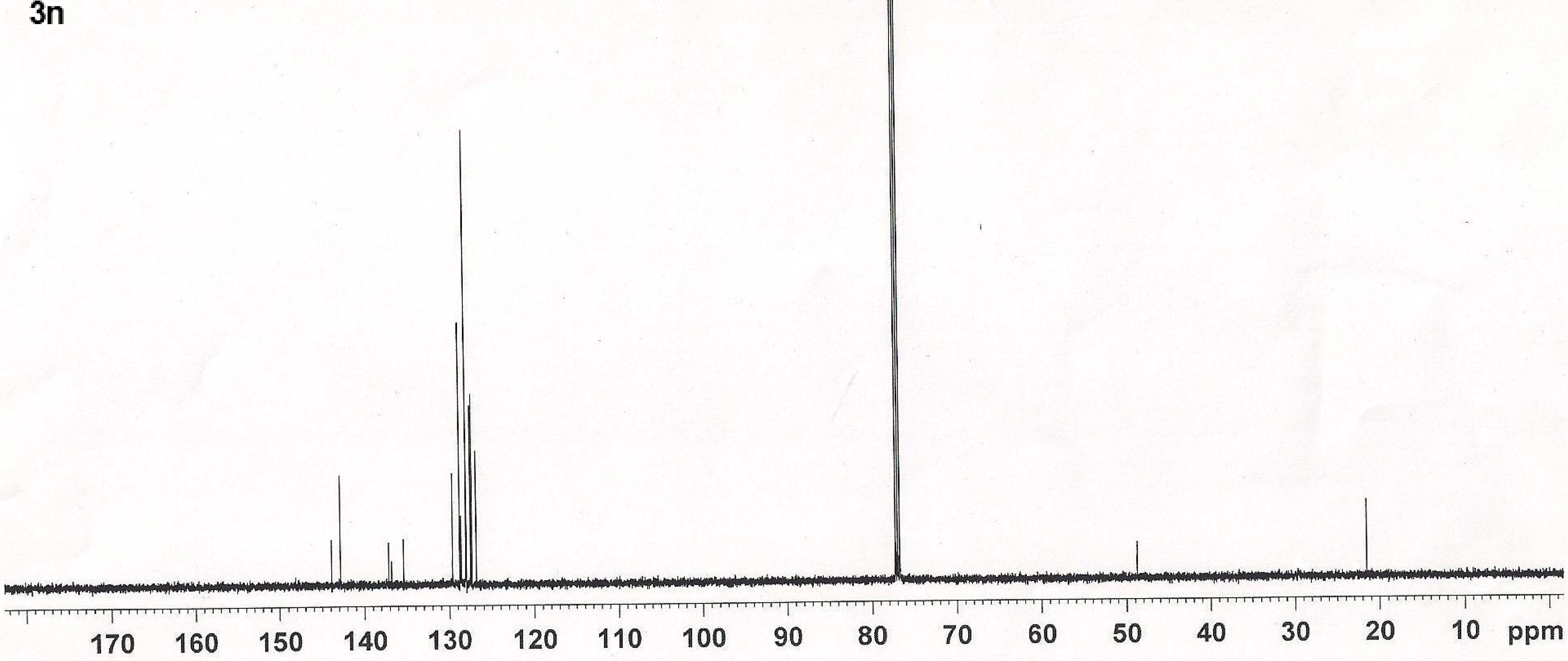

Supplement: File 1 — Analytical and spectroscopic data for compounds 3a–3j, 3ka, 3kb and 3l–3n. [file Beilstein_J_Org_Chem-09-1045-s001.pdf]
